# Left ventricular rigor mortis interferes with postmortem aortic root geometry

**DOI:** 10.1007/s00414-025-03409-1

**Published:** 2025-01-21

**Authors:** Jan Michael Federspiel, Karen B. Abeln, Frank Ramsthaler, Thomas Tschernig, Peter H. Schmidt

**Affiliations:** 1 Institute for Legal Medicine, Faculty of Medicine, Saarland University, Campus Homburg, Building 49.1, Kirrberger Straße 100, 66421 Homburg/Saar, Germany; 2 Department of Cardiac Surgery, Saarland University Medical Center, Homburg/Saar, Germany; 3 Institute of Anatomy, Faculty of Medicine, Saarland University, Campus Homburg, Homburg/Saar, Germany

**Keywords:** Aortic valve, Aortic root, Cardiac geometry, Postmortem examination, Left ventricular rigor mortis, Postmortem interval

## Abstract

Aortic regurgitation is a common valve disease and can be caused by delineated findings such as fenestrations or hardly discernible alterations of the aortic root geometry. Therefore, aortic regurgitation can be a challenging diagnosis during an autopsy. Cardiac surgeons, however, are confronted with comparable problems during surgery and have developed a refined knowledge of the anatomy of the aortic root including its geometry. Transferring this knowledge from the operating room to the dissection would further complement the panel of postmortem diagnostic tools. To foster translation of the clinical anatomy, the present study assessed the impact of postmortem peculiarities (i.e. myocardial rigor mortis, putrefaction) that might influence aortic root geometry. The aortic root geometry was described by aortic perimeters (basal, sinus, sino-tubular junction, and ascending aorta), effective height (distance from the cusp’s free margin to its nadir), geometric height (cusp height), commissural height (distance from the base of an interleaflet triangle to the end of a commissure), and length of the ascending aorta. Data from 140 cases were analyzed (linear regression, comparative testing). Myocardial rigor mortis was associated with smaller basal rings. Weak positive correlations between the duration of the postmortem interval and aortic root dimensions were observed. In summary, postmortem peculiarities, especially the myocardial cadaveric rigidity, influence postmortem aortic root geometry. Despite these circumstances, the current study demonstrates that aortic root geometric assessment, including effective height, is feasible in a postmortem setting. Further studies are needed to elaborate on aortic root geometry as a diagnostic tool in a necropsy setting.

**Supplementary Information:**

The online version contains supplementary material available at 10.1007/s00414-025-03409-1.

## Introduction

Legal Medicine serves as an interface between Medicine and Law [1]. By that, Legal Medicine experts are involved in the workup of non-natural [2] and unclear deaths [3, 4]. The latter in particular includes cases of unexpected deaths [5]. In these cases, it is the primary task of Legal Medicine experts to thoroughly search for injuries hinting at foreign violation or foul play. If evidence of an external impact on the causal chain leading to death cannot be identified, the assessment of a fatal disease further supports the finding of a natural chain of death. In cases of an unclear and/or unexpected death, a cardiac disease is frequently found as the cause of death [6, 7]. Such a sudden cardiac death (SCD; summary of all abbreviations in Supplemental File I - Appendix A) is defined ‘as the natural unexpected death of unknown or cardiac cause’ ( [8], page 15) in an autopsy setting. Thus, to establish the diagnosis either all non-cardiac causes must be precluded or a cardiac pathology has to be identified. Correspondingly, subsequent investigations, such as toxicology, are required in the assessment of SCDs [5, 9, 10]. However, the exclusion of all non-cardiac causes, especially those associated with minor or none gross findings such as excited delirium [11], is hardly possible without comprehensive and time consuming subsequent analyses. Thus, especially the gross, i.e. swift and time-efficient, identification of a morphological substrate of SCD during autopsy is of particular practical benefit in Legal Medicine as both jurisdiction and law enforcement require at least a fast initial (preliminary) expert opinion to support their investigations.

Due to its broad definition SCD is a general term summarizing a variety of pathologies and pathophysiologies. Some of them are associated with grossly delineated findings such as acute coronary occlusion [12]. Others, especially functional phenomena, such as arrhythmias, remain diagnostic challenges and can require subsequent comprehensive analyses, such as genetic testing, due to the lack of macroscopic findings [13]. In this context, valvular heart disease can present a particular postmortem diagnostic challenge as it may be associated with arrhythmias [14, 15] and obvious surrogates of valve malfunction (e.g. perforations [16]), may be absent. Due to the complex functional anatomy of heart valves, the absence of such surrogates, does not allow for the assumption of a functionally competent valve. For example, aortic regurgitation (AR) can be caused by discrete geometric alterations of the aortic root (AoR) [17–20], i.e. the complete functional unit of the aortic bulb, including the aortic valve (AV) cusps [21]. Thus, heart valve disease remains a diagnostic challenge during autopsy. This is underpinned by discrepancies between postmortem and clinical studies outlined in the following paragraphs.

With regard to valvular heart disease in general, a large clinical cohort study revealed that even in the clinical setting of elderly primary care, valvular heart disease has not been detected before in around 50% of elderly individuals [22]. Despite such cases of unrecognized valvular heart disease, clinical studies found deaths related to valvular heart disease in around 0.8% of all-cause mortality and approximately 2.4% of cardiovascular deaths [23]. In contrast, postmortem studies suggest that SCD due to valvular heart disease is rare [24, 25]; only about 1.2% of SCD cases were found to be associated with valvular heart disease [25].

This discrepancy between clinical and postmortem observations is particularly striking regarding AR. Clinically, AR can be found in approximately 8.5% of females and 13% of males [26] with mortality rates of up to 34% under conservative treatment [27]. In contrast, postmortem investigations only identified AR in 4 out of 5869 SCD victims [25], suggesting that AR is underestimated in autopsy studies. Additionally, there is a lack of in-depth assessment of functional phenomena associated with AR comparable to what is known about the mitral valve (MV; [14, 15]). An example would be that a thorough examination of functional AV anatomy, which includes AoR geometry, is not included in postmortem recommendations [5]. Consequently, the threshold to be considered a certain or highly probable SCD substrate regarding the AV is seemingly high [5], as for the native AV only a bicuspid AV (BAV) with aortic dissection is assumed as a *certain* SCD substrate at postmortem investigation whereas AR associated with a dilated aortic annulus is only rated as an *uncertain* substrate [5]. Overall, current routine measures of AR due to alterations in AoR geometry may underestimate SCD associated with AR in a postmortem setting focused on morphological substrates of SCD such as Legal Medicine.

Clinical anatomy may help to close this gap, and could further expand the postmortem diagnostic panel. Cardiac surgeons recognized the importance of AoR geometry for AV function early on (e.g [17, 18, 28]). and established the concept of effective height (eH) [29–32] for AoR assessment during surgery, i.e. without circulation. Effective height is the height difference between the cusps’ central free margins and cusp insertion lines [30]. Studies assessing this AoR measure in a postmortem setting, are lacking (details Supplemental File I – Appendix B).

This study therefore aims to lay the foundation for transferring the refined knowledge of clinical anatomy of AoR geometry to a necropsy setting. It is particularly important in a Legal Medicine setting as the interaction with jurisdiction and law enforcement requires safe but also swift and time-efficient diagnoses. A particular focus on postmortem peculiarities was applied and it was investigated how increasing duration of the postmortem interval (PMI) and left ventricular (LV) rigor mortis affect AoR geometry. Herein, the present study follows recent requests, also involving Legal Medicine Societies such as the Swiss Society of Legal Medicine, to “build bridges” between the clinical sector and Legal Medicine in order to improve diagnostic performance [3]. Thus, the long-term goal of subsequent studies will be to establish AoR geometry as a diagnostic tool in the postmortem setting in order to improve the identification of SCD surrogates at autopsy in Legal Medicine.

## Materials and methods

### Assessed parameters, data acquisition, and statistics

**Fig. 1:**
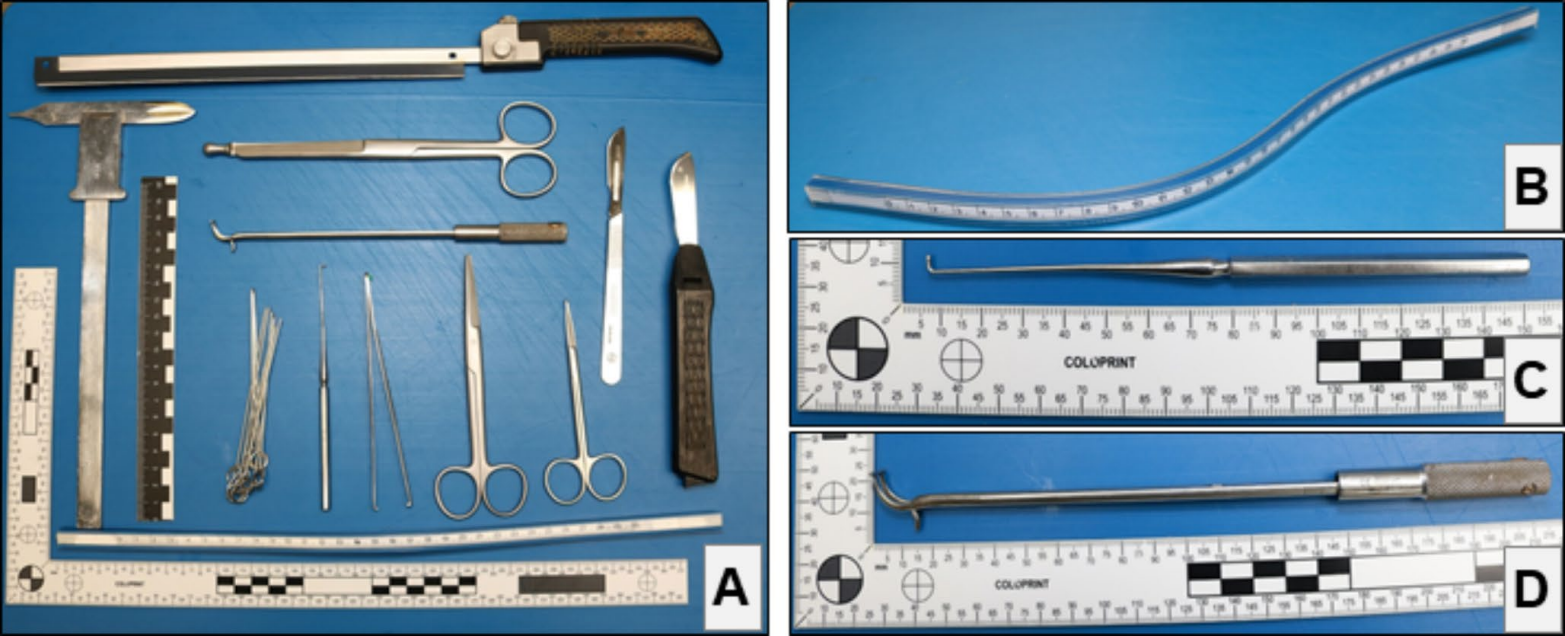
Set-up and instruments **A**: Shown are all the instruments used to assess the hearts, including different knives, hooks, scissors, rulers, and needles. Not pictured is the Canon EOS 850D with a Canon EF-S 18–55 mm lens, and Speedlite 430EX III-RT flash used for photographic documentation. The needles were used to stretch the heart on a sponge in case rigor mortis complicated the measurements. Elastic rulers (e.g. **B**) were used to facilitate measurements following the actual course of the respective structure, for example, the complex shape of the tricuspid valve annulus [62]. A small nerve and vessel hook (**C**) was used to assess the heart valves before opening the heart. The so-called *Caliper* (**D**; Fehling Instruments, Karlstein, Germany; manufacturer homepage: https://www.fehling-instruments.de/) was used to measure the effective height (eH). **Abbreviations**: eH – effective height

**Fig. 2:**
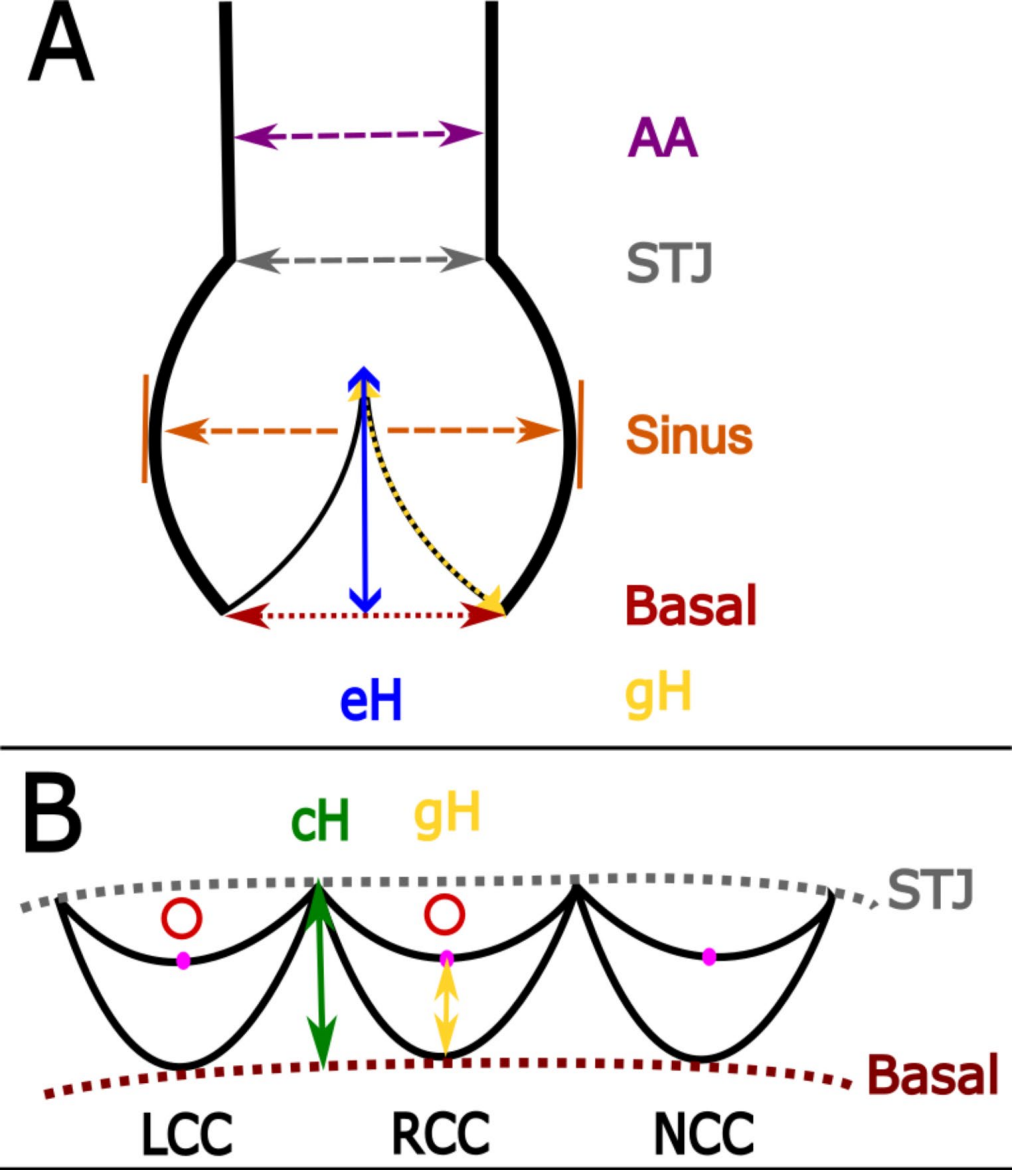
Schematic drawing of aortic root measurements **A**: Schematic drawing of the proximal ascending aorta (AA) in a frontal dissection plane. Each arrow indicates a measurement performed. Purple – ascending aortic (AA) perimeter approximately 1 cm cranial to the aortic valve (AV) commissures. The measurement of the AA length is not displayed. Grey – sino-tubular junction (STJ) perimeter where the aortic bulb transitions to the tubular AA. Orange – measurement of the Valsalva sinus perimeter at the outermost circumference of the aortic bulb. Dark red – AV virtual basal ring perimeter (details see Fig. 2-B). Blue – effective height (eH) measurement. The eH is defined as the height difference between the cusp’s central free margin and the cusp insertion lines [30]. It has been measured using Fehling’s caliper (details see Fig. 1). With that, it is important to align the instrument along the axis of the left ventricular (LV) outflow tract [32] to allow for correct measurements. These measurements must be done before opening of the heart and aortic root (AoR). Details on eH can be found in [30, 32, 63]. Yellow – geometric height (gH). The gH was measured along the pars ventricularis of each cusp from the nodulus Arantii perpendicular to the nadir of the respective cusp (see Fig. 2-B). Details regarding gH can be found in [31, 32]. **B**: Schematic drawing of the longitudinally opened AoR. The STJ is indicated by the dotted grey line. The virtual basal ring, the line connecting the nadirs of all three cusps, is displayed as a dotted dark red line. Bright red indicates the two coronary ostia. The pink dots represent the Arantii’s nodulus of each cusp. The gH (yellow) is measured as described in Fig. 2-A. The green line shows the measurement of the commissural height (cH). - **General Note**: Details on the clinical anatomy of the aortic root can be found in [50]. In case the coronary arteries are opened longitudinally, the AoR measurements should be done before coronary preparation to avoid any potential interference with the geometry. - The figure was generated using Inkscape 1.3.2 (091e20e, 2023-11-25, custom). - **Abbreviations**: AA – ascending aorta; AoR – aortic root; AV – aortic valve; cH – commissural height; eH– effective height; gH – geometric height; LCC– left coronary cusp; LV – left ventricle; NCC – non-coronary cusp; RCC – right coronary cusp; STJ – sino-tubular junction

**Fig. 3:**
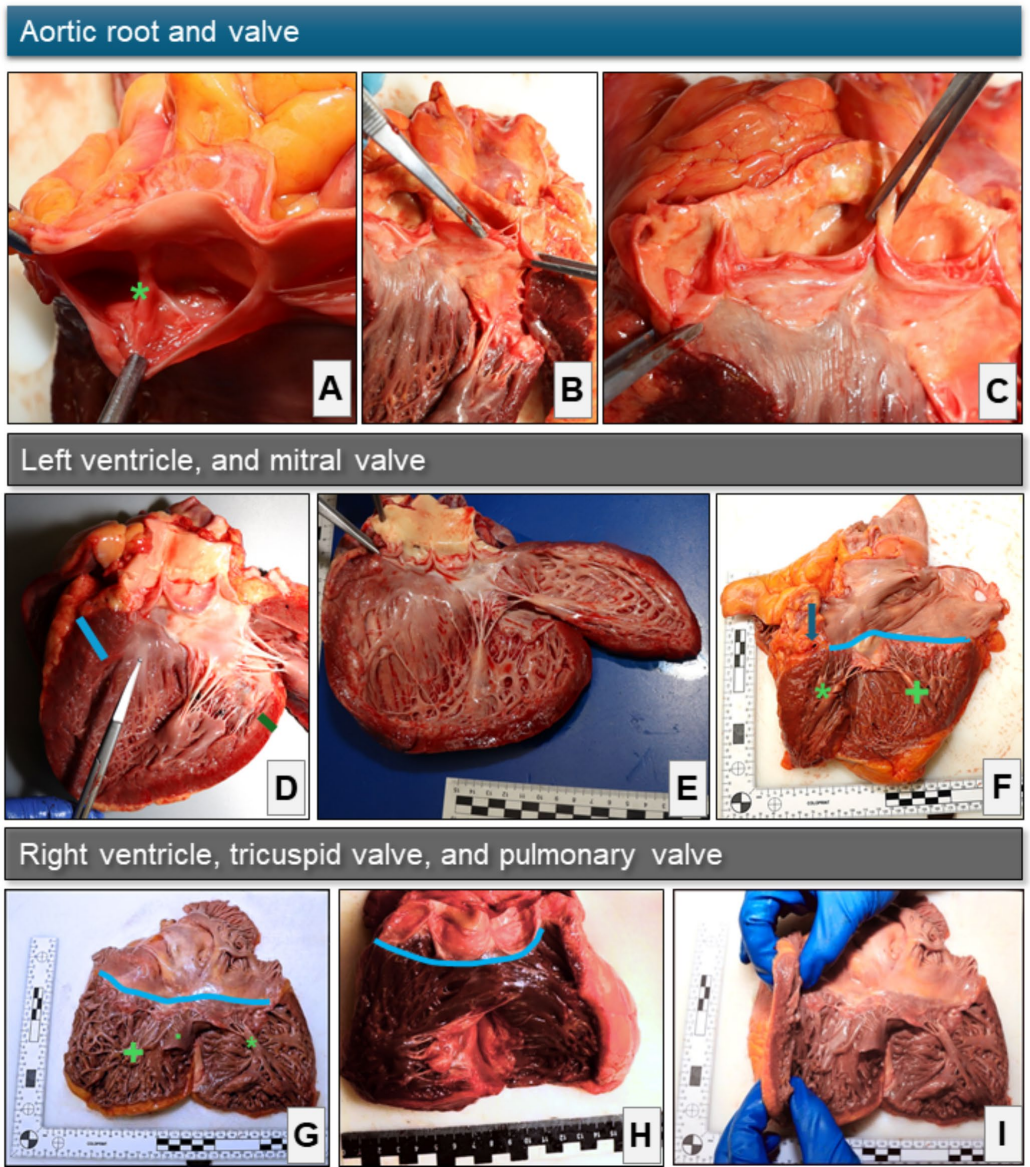
Assessment of the heart Provided are examples of morphologic assessments of the aortic root (AoR) and the measurements of the non-aortic valve (AV) perimeters (i.e. tricuspid valve, pulmonary valve, and mitral valve, MV). The morphometric assessment of the AoR is displayed in Fig. 2. - When using the inflow-outflow-preparation technique, particular attention was paid to the perpendicular dissection of the myocardium to avoid incorrect measurements of wall thickness. The photographs shown in this figure were edited using Microsoft^®^ Office 365 tools. **A**: Close-up photograph of the fused cusp of a bicuspid aortic valve (BAV) with a fusion of the left coronary (LCC) and right coronary cusp (RCC). The green asterisk (*) marks the center of the raphe restricting the cusp. **B**: Close-up photograph of the left ventricular (LV) outflow tract and AoR after longitudinal opening along the outflow tract axis. The left forceps grab the non-coronary cusp (NCC), and the right forceps hold one-half of the LCC. There is a fenestration in the NCC that extends to the commissure between the NCC and LCC. The presence or absence of fenestrations extending toward a commissure was captured along with their localization. **C**: Shown is the same AoR as in B. The left forceps hold the LCC nadir and the right forceps grab the commissure between the RCC and NCC. Notably, this commissure does not reach the same height as the other two commissures. In addition, the upper parts of the commissure are fused, as the cusps are not visible up to the cranial end of the commissure. Therefore, there is a short partial fusion of the RCC and NCC. The presence or absence of (partial) fusions was indicated together with their localization and, if measurable, their length. **D**: Example of myocardial rigor mortis. Overview of the LV outflow tract after longitudinal opening. The light source was placed on the right side. Note the shadow cast by the interventricular septum (IVS) on the left side of the image, indicating a convex shape despite the opening of the heart. The scissors are aligned with the LV outflow tract axis, with the tip at the level of the subaortic IVS. Next to the tip is a small septal bulge, a phenomenon frequently observed in LV rigor mortis. The thickness of IVS was measured 1 cm apical to the basal ring (approximated by the light blue line). A measurement of the LV posterior wall 1 cm apical to the MV annulus was noted as ‘LV wall thickness’ (approximated by the green line). **E**: Example of a flaccid and protruding LV. Without further consideration of the valvular rings and the underlying disease, this phenomenon was termed ‘ventricular dilatation’. **F**: Overview of the left atrium and the LV inflow tract after opening of the heart. The asterisk (*) indicates the anterior papillary muscle. The posterior papillary muscle is highlighted by the plus (+). The arrow points to the cross-section of the circumflex artery on the anterior LV wall. The blue line approximates the course of the MV annulus. **G**: Right atrium and right ventricular (RV) inflow tract with an overview of the tricuspid valve apparatus. The blue line indicates the course of the tricuspid valve annulus. The asterisk (*) marks the anterior papillary muscle. The dot (.) highlights the septal papillary muscle. The plus (+) highlights the posterior papillary muscle. **H**: Overview of the apical and outflow tract portion of the RV together with the root of the pulmonary trunk. The pulmonary valve perimeter was measured at the level of the virtual basal ring connecting the nadirs of the cusps (blue line) in analogy to the AV. The RV wall thickness was measured in the outflow tract 1 cm apical to the virtual basal ring of the pulmonary valve. **I**: Overview of the right atrium and RV inflow. Shown is an example of marked RV hypertrophy with coarse and thick trabeculation. **Abbreviations**: AoR – aortic root; AV – aortic valve; BAV – bicuspid aortic valve; cH – commissural height; eH – effective height; gH – geometric height; IVS – interventricular septum; LCC – left coronary cusp; LV – left ventricle / left ventricular; MV – mitral valve; NCC – non-coronary cusp; RCC – right coronary cusp; RV – right ventricle / right ventricular; STJ – sino-tubular junction

The present manuscript describes an explorative morphological and morphometric study macroscopically assessing non-fixated hearts. Figure 1 displays the instruments used. The data collected and measurements taken are explained, defined, and summarized in Table 1, Fig. 2, and Fig. 3. The AoR geometry was described by the virtual basal ring perimeter (basal), largest Valsalva sinus perimeter (Sinus), sino-tubular junction perimeter (STJ), tubular ascending aorta (AA) perimeter and length, eH, geometric height (gH), and commissural height (cH; definitions see Table 1, Fig. 2, and Fig. 3).

**Table 1:** Parameters assessed and data collected

Case and individual characteristics	
Postmortem Interval (PMI) [h] ^1^	Grade of Putrefaction ^2^
Sex	Age [years]
Body height [m]	Body weight [kg]
Body surface area (BSA, Mostellar formula) [m²]	Body mass index (BMI) [kg/m²]
Cause of death^3^	
***Gross assessment of the heart*** ^***4***^	
Presence/absence LV rigor mortis ^5^	HW [g]
Predicted HW according to [35]	Presence/absence of AA aneurysm ^6^
Wall thickness ^7^ [mm]: - LV - right ventricle (RV) - IVS	Wall thickness ratio (modified adapted from [64]): - IVS to LV - IVS to RV - LV to RV
Visual impression of LV dilation ^8^	
***Morphometric assessment of the heart***	
Non-aortic ring perimeters [mm]: - tricuspid valve - pulmonary valve - MV	Normalized (body height) ring perimeters (modified adapted from [64]).
AA dimensions [mm]: - Length ^9^ - diameter (1 cm above the commissures)	Normalized (body height) aortic dimensions (modified adapted from [33]).
AoR perimeters [mm]: - virtual basal ring - largest perimeter Valsalva sinus - STJ	Normalized (body height) AoR parameters in analogy to AA dimensions.
Further AoR parameters (if measured [mm]): - visual coaptation - AV morphology ^10^ - presence/absence of fenestrations extending towards a commissure - eH, gH, and cH	Normalized (body height) AV parameters to body height in analogy to AA dimensions as, for example, gH and body height correlate in tricuspid AVs (TAV) [31].

Due to the rigorous data preparation, no missing values were encountered. **Annotations**: ***1*** - If the time point of death was known (e.g. death in the intensive care unit), an ‘exact’ PMI was calculated between the time of death and the beginning of the autopsy. If the exact time of death was unknown, the time at which the person was last seen alive was used to conservatively estimate the maximum possible PMI. In such cases, an ‘estimated’ PMI was reported. -***2***- For the present study, putrefaction was described as follows: *Mild* – Putrefaction largely limited to the body surface; *Moderate* - More than mild putrefaction but not yet impairing organ structure; S*evere* - Putrefaction that visibly altered the organ structure. -***3***- Cause of death was classified for the statistical analyses as follows: unclear cause of death; cardiovascular cause of death; intoxication; non-cardiac disease; and traumatic death. A case-by-case description of the cause of death is shown in Supplemental File I – Appendix C. -***4***- The hearts were roughly analyzed according to the Institute’s standard routine (inflow-outflow preparation). HW was measured after the opening of the cardiac chambers and removal of postmortem thrombi. -***5***- LV rigor mortis was classified as ‘present’ if the heart felt like a contracted muscle on palpation, maintained a round or conical shape despite LV opening, and required some ‘breaking’ of the LV outflow tract to achieve a more plane surface for measurements. If these features were not observed, LV rigor mortis was categorized as ‘absent’. -***6***- An AA aneurysm was categorized as present if the AA was markedly thicker than the pulmonary trunk. -***7***- Wall thicknesses were measured 1 cm apical to the valvular plane. -***8***- Independent of the annular measurements, the LV was classified as ‘dilated’ if the LV extended limply beyond the outermost point of the MV annulus before the opening of the heart. -***9***- AA length was measured from the STJ along the convexity of the AA to the orifice of the brachiocephalic trunk. -***10***- The AV morphology was defined based on the commissures [65]. A TAV was present if all three commissures were of equal height. In a BAV, one commissure was restricted in height. Thus, if partial fusion of two cusps without commissural height restriction was observed, this was classified as degenerative fusion in a TAV. If partial fusion with cH restriction was observed, the valve was classified as BAV [66]. Further details on the measurements performed and the definitions used can be found in Figs. 2 and 3. **Abbreviations**: AA – tubular ascending aorta; AoR – aortic root; AV – aortic valve; BAV – bicuspid aortic valve; BMI – body mass index; BSA – body surface area; cH – commissural height, eH – effective height; gH – geometric height; HW – heart weight; IVS – interventricular septum; LV – left ventricle; MV – mitral valve; PMI – postmortem interval; STJ – sino-tubular junction; TAV – tricuspid aortic valve

Data were collected by one investigator (first author) between August 2021 and July 2024 during routine autopsies (Legal Medicine). The inclusion criterion age (i.e. at least 18 years). Exclusion criteria were time pressure (e.g. increased public interest in the case) or not clearly discernible AoR structures (e.g. trauma). Applying these criteria, data were collected in 196 cases from 320 autopsies performed by the investigator. Due to the variety of factors that could interfere with the measurements (e.g. body height [33]) and an assumed postmortem variance (e.g. varying PMI associated with different degrees of putrefaction), only complete data sets were forwarded for statistical analysis. Data preparation was done using Microsoft^®^ Excel (Office 365, Version 2406 Build 17726.20160 Click-to-Run). A total of 140 cases were forwarded for statistical analyses (R Version 4.4.1; RStudio Version 2024.04.02 Build 764). The final anonymized data set (Supplemental File I), the R code (Supplemental File I), and comments on the data preparation (Supplemental File IV) can be found in the supplement.

If applicable, continuous data were normalized to the body height. The normalized values were used for statistical analyses. Normal distribution was assumed. Continuous variables were described using mean (M) and standard deviation (SD). Non-continuous variables were described using absolute and relative frequencies. If not declared otherwise, a percentage refers to the cohort (*n* = 140). Due to the exploratory nature of the study and the associated high number of statistical tests, a significance level of α = 0.01 was chosen. Following current recommendations, the authors attempted to avoid using ‘(statistically) (non)significant’ [34].

**Fig. 4:**
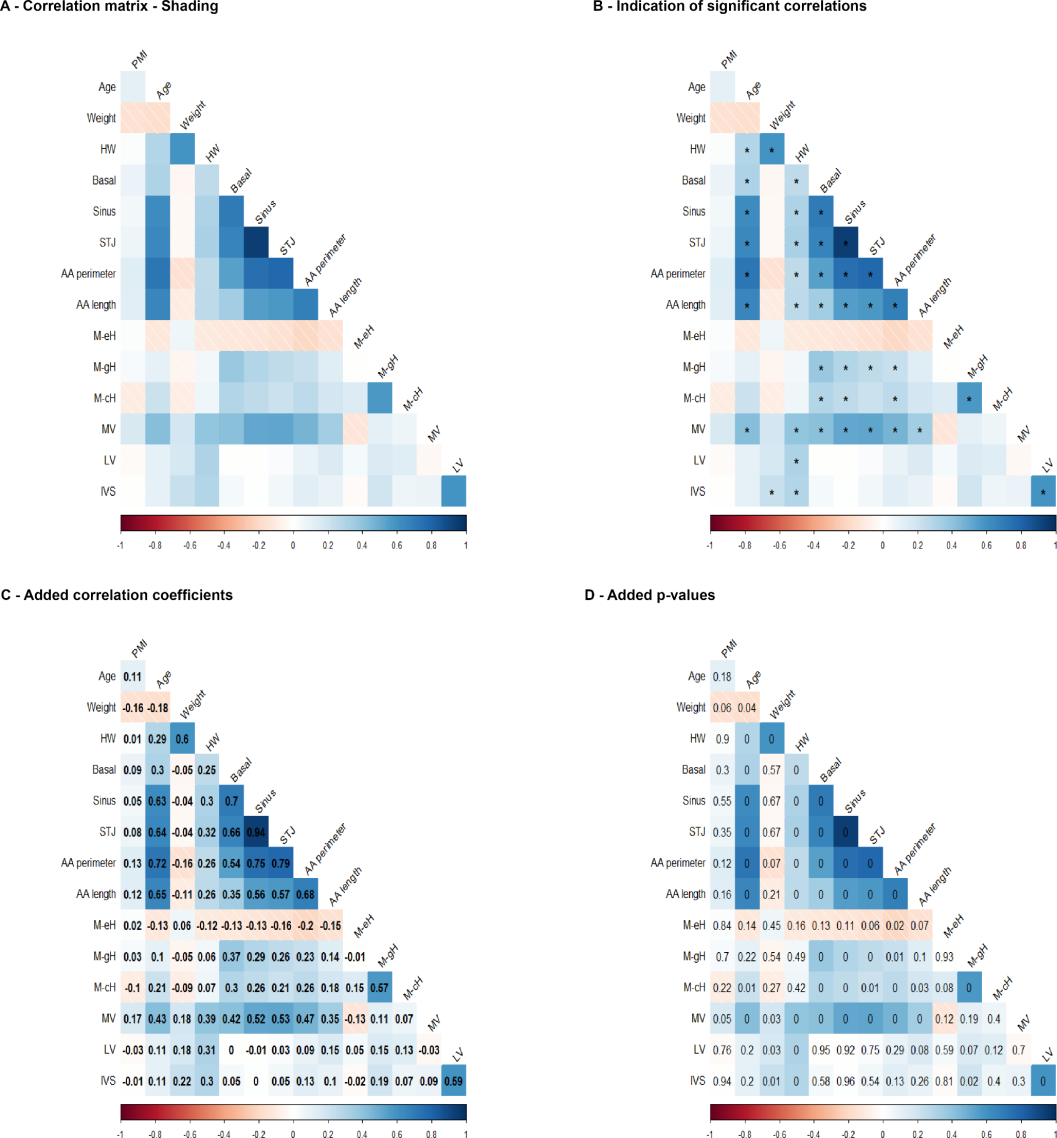
Correlation matrix From A to D, the blue color indicates positive correlations, whereas the red color indicates negative correlations. The color intensity encodes the strength of the correlation. **A**: the plane correlation matrix. **B**: correlations with a p<0.01 are marked with an asterisk (*). **C**: matrix with added correlation coefficients (ρ). **D**: p-values for each correlation. Note that this figure shows correlation coefficients and p-values rounded to two decimal places. Therefore, values of 0.01 may be the residuals of values that are in fact <0.01. The non-rounded values on which the matrix is based can be found in the text and the appendix (Supplemental File I). Abbreviations: AA – ascending aorta; HW – heart weight; IVS– interventricular septum; LV– left ventricle; M-cH– mean commissural height; M-eH – mean effective height; M-gH – mean geometric height; MV – mitral valve; STJ – sino-tubular junction

A matrix of linear correlations (Spearman, i.e. ρ indicates correlation coefficients) was created to screen for factors potentially influencing the postmortem AoR geometry. The matrix encompassed the parameters describing the AoR geometry and parameters that may potentially interfere with the postmortem shape of the left heart (i.e. PMI, body weight, heart weight (HW), MV, perimeter, LV wall thickness, and thickness of the interventricular septum (IVS)). Applying this approach led to obvious correlations, for example, a correlation between LV wall thickness and HW. Such obvious correlations are not further described, but only the correlations related to the analysis of interest (i.e. AoR geometry) are described further. The complete correlation matrix, however, is shown in Fig. 4. All p-values and correlation coefficients calculated are reported in Supplemental File V.

In addition, comparative testing was applied searching for differences regarding the AoR geometry in the context of potential influential factors, i.e. grade of putrefaction, cause of death, and the presence/absence of different findings: LV rigor mortis, visual hints of AA aneurysm, visual AV coaptation, cusp fusion, and fenestrations extending towards a commissure. For dichotomous comparisons, the Wilcoxon test (W) was applied. The resulting 48 p-values were adjusted using the Benjamini-Hochberg method. In more than dichotomous comparisons, the Kruskal-Wallis (KW) test was employed to screen for trends toward differences. If the KW’s p-value was < 0.01, the Games-Howell-Test was applied for post-hoc testing. P-values during post-hoc testing were adjusted by applying Tukey’s method.

### Study cohort

**Fig. 5:**
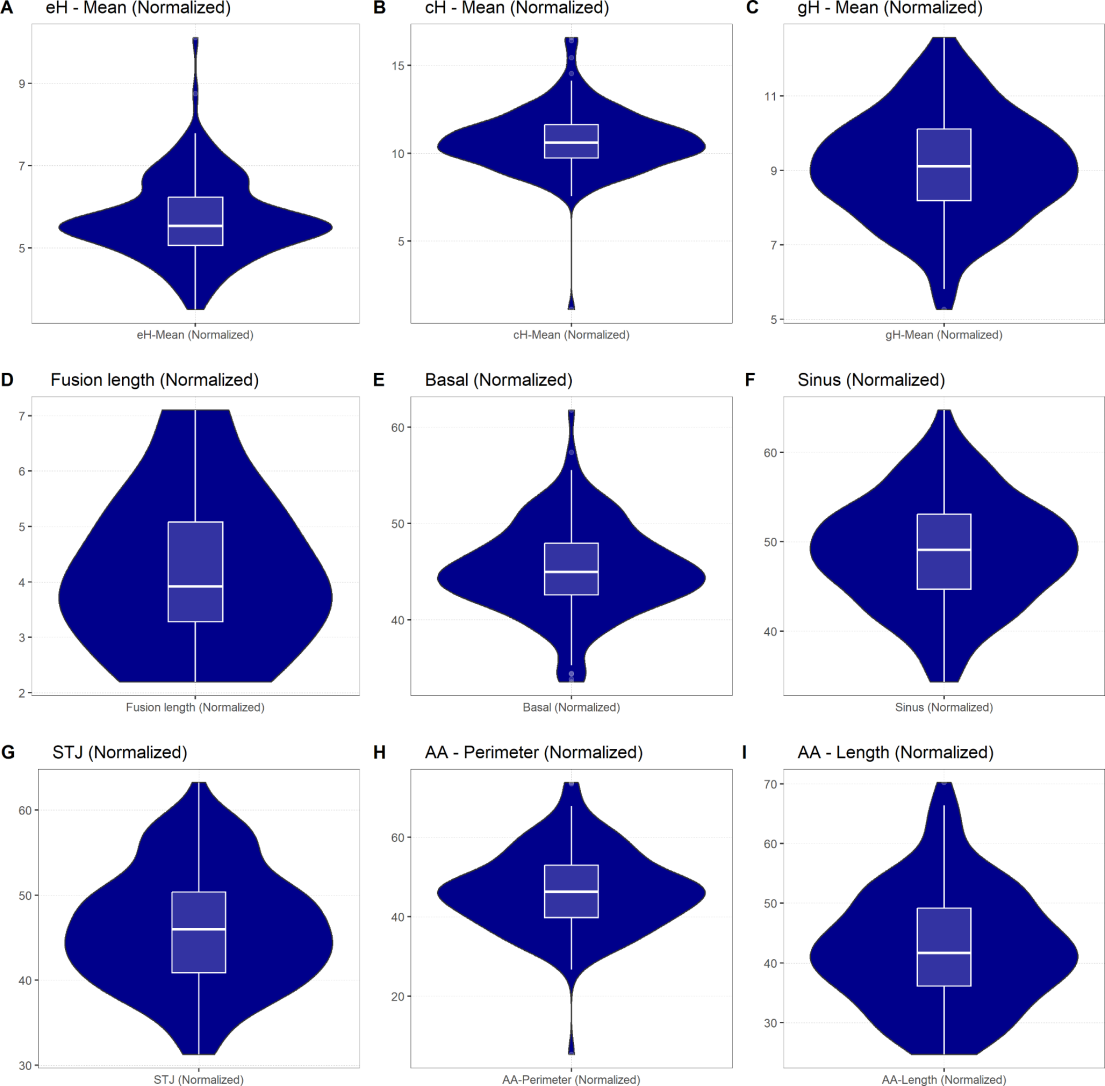
Violin plots describing the postmortem AoR geometry Each violin plot (dark blue area) resembles the distribution of the respective parameter. The wider the area, the more individuals had the respective value. The box plot (white) within each violin shows the descriptive statistics: Each box covers the interquartile range of the respective parameter (i.e. 25th percentile at the top, 75th percentile at the bottom). The line within the box resembles the median. Each whisker ends above or below the first and third quartile plus/minus 1.5 times the interquartile range. Values outside these borders are displayed as larger dots, i.e. as outliers. **Abbreviations**: AA – tubular ascending aorta; AoR – aortic root; cH – commissural height; eH– effective height; gH – geometric height; LCC – left coronary cusp; NCC – non-coronary cusp; RCC – right coronary cusp; STJ – sino-tubular junction

The majority of individuals were male (*n* = 102/140, 72.86%) with a mean age of 60.35 years (SD 18.87). The mean body height was 171.06 cm (SD 10.12), and the mean body weight was 81.21 kg (SD 23.7; BMI [kg/m²]: M 27.68, SD 8.2). In 92 cases (65.71%), the ‘exact’ PMI was calculable based on the information provided by the police (time between point of death to autopsy). In 48 cases (34.29%), the PMI was estimated (time between last seen alive to autopsy). Overall, the mean PMI was 130.92 h (SD 169.56). In most cases, mild putrefaction (*n* = 122, 87.14%) was observed. In the majority of cases, the cause of death could be determined (*n* = 120, 92.14%), and traumatic death was observed most frequently (*n* = 43, 30.71%) (see Supplemental File I – Appendix C). The mean HW was 439.27 g (SD 128.79). In 27 cases (19.29%), the measured HW was above the upper limit of the 95%-confidence interval of the HW predicted by applying established regression formulas [35]. LV rigor mortis was present in 42 cases (30%). Signs of ventricular dilatation were observed in 57 cases (40.71%) and of AA aneurysm in 45 cases (32.14%). Most individuals had a tricuspid AV (TAV, *n* = 134, 95.71%). BAV morphology was observed in 6 instances (4.29%). No morphologies other than BAV and TAV, for example, unicuspid or quadricuspid, were observed. Descriptive statistics are presented in detail in Tables 2 and 3, and Fig. 5. Further descriptive statistics can be found in Supplemental File I – Appendix D.

**Table 2:** Descriptive statistics of non-continuous variables

	*N*	%
Sex: Male / Female	102 / 38	72.86 / 27.14
***Cause of death***		
Unclear cause of death	11	7.86
Cardio-vascular cause of death	39	27.86
Non-cardiac disease	35	25
Intoxication	12	8.57
Traumatic	43	30.71
***Putrefaction*** ^***1***^		
Mild	122	87.14
Moderate putrefaction	18	12.86
***Heart and ascending aorta***		
Gross hint for AA aneurysm	45	32.14
LV rigor mortis present	42	30
Presence / Absence hints for left ventricular dilation	57 /83	40.71 / 59.29
HW exceeding the upper limit of 95%-confidence interval	27	19.29
***Aortic valve***		
Visual coaptation	124	88.57
Fusion		
Absent / Present	127 / 13	90.71 / 9.29
Partial / Complete	11 / 2	7.86 / 1.43
With / Without restriction of commissural height	6 / 7	4.29 / 5
of RCC / NCC	10	7.14
of RCC / LCC	2	1.43
of LCC / NCC	1	0.71
Tricuspid / bicuspid aortic valve	134 / 6	95.71 / 4.29
Fenestration extending to the commissure	13	9.29
towards non-height restricted LCC / NCC commissure	2	1.43
towards non-height restricted RCC / NCC commissure	3	2.14
towards non-height restricted LCC / RCC commissure	8	5.71
in LCC / in RCC	3 / 2	2.14 / 1.43
in NCC / in fused cusp	4 / 3	2.86 / 2.14
In both, LCC and RCC	1	0.71

For details on the parameter definitions and measurements see Table 1; Fig. 2. **Annotations**: 1- Severe putrefaction was not observed. **Abbreviations**: AA – ascending aorta; HW – heart weight; LCC – left coronary cusp; LV – left ventricle; NCC – non-coronary cusp; RCC – right coronary cusp

**Table 3:** Descriptive statistics of continuous variables

	M	SD
PMI [h]		
‘exact’ (available in *n* = 92, 65.71%)	86.24	53.95
‘estimate’ (available in *n* = 48, 34.29%)	216.55	260.77
overall (i.e., exact and estimate combined, *n* = 140)	130.92	169.56
***Basic characteristics***		
Age [years]	60.35	18.87
Body height [cm]	171.06	10.12
Body weight [kg]	81.21	23.7
BMI [kg/m²]	27.68	8.2
BSA [m²]	1.95	0.3
***Heart in general and heart weight***		
Wall thicknesses LV [mm] / IVS [mm] / RV [mm]	15.01 / 15.49/ 5.13	2.72 / 3.38/ 2.62
Ratios: LV/RV / IVS/RV / IVS/LV	3.37 / 3.53 / 1.04	1.2 / 1.3 / 0.22
Tricuspid valve perimeter [mm] – measured/normalized	127.45 / 74.58	17.14 / 9.59
Pulmonary valve perimeter [mm] – measured/normalized	83.97 / 49.12	12.97 / 7.24
MV perimeter [mm] – measured/normalized	111.04 / 65.22	72.51 / 45.51
HW measured [g] / predicted - Point [g]	439.27 / 399.67	128.79 / 70.82
HW predicted - Plus 1 standard deviation	458.67	81.41
HW predicted - Upper limit 95% Confidence Interval	502.02	91.43
***AoR and AV – measured/normalized***		
Basal [mm]	77.33 / 45.26	8.46 / 4.69
Sinus [mm]	83.5 / 48.9	10.52 / 6.15
STJ [mm]	78.71 / 46.09	11.73 / 6.8
AA – perimeter [mm]	79.21 / 46.52	16.20 / 10.07
AA - length [mm]	72.75 / 42.60	16.66 / 9.69
eH – RCC [mm]	9.76 / 5.74	1.69 / 1.11
eH – LCC [mm]	9.72 / 5.72	2.07 / 1.3
eH – NCC [mm]	9.83 / 5.77	1.84 / 1.13
eH – Fused cusp [mm]	6.83 / 3.84	0.98 / 0.61
M-eH per case [mm]	9.71 / 5.7	1.5 / 0.99
gH - RCC [mm]	15.56 / 9.12	2.6 / 1.43
gH - LCC [mm]	15.54 / 9.1	2.67 / 1.48
gH - NCC [mm]	15.53 / 9.09	2.61 / 1.45
gH - Fused cusp [mm]	16.33 / 9.11	3.72 / 1.93
M-gH per case [mm]	15.57 / 9.1	2.62 / 1.44
cH – RCC/LCC [mm]	18.29 / 10.72	3.15 / 1.86
cH– RCC/NCC [mm]	18.19 / 10.66	3.31 / 1.95
cH – LCC/NCC [mm]	18.34 / 10.75	3.06 / 1.8
M-cH per case [mm]	18.27 / 10.71	3.05 / 1.81
Fusion length [mm]	7 / 4.19	2.61 / 1.48

For details on the parameter definitions and measurements see Table 1; Fig. 2. **Abbreviations**: AA – tubular ascending aorta; AoR – aortic root; AV – aortic valve; BMI – body mass index; BSA – body surface area according to the Mostellar formula; cH – commissural height; eH – effective height; gH– geometric height; HW– heart weight; IVS – interventricular septum; LCC– left coronary cusp; LV – left ventricle; M – mean; MV – mitral valve; NCC– non-coronary cusp; PMI – postmortem interval; PV– pulmonary valve; RCC – right coronary cusp; RV – right ventricle; SD – standard deviation; STJ– sino-tubular junction; TV – tricuspid valve

## Results

Results are summarized in Fig. 4; Table 4. Further details regarding the correlation analysis can be found in Supplemental File V.

**Table 4:** Comparative analysis

	LV rigor mortis ^W^							Putrefaction grade ^W^							
(M / SD)	Present	Absent		*p*		*p* adj.		Mild	Moderate		*p*			*p* adj.	
*M-eH*	6 / 1.23	5.57 / 0.83		0.1084		0.2262		5.74 / 1.01	5.44 / 0.81		0.2814			0.4502	
*M-cH*	10.71 / 1.59	10.71 / 1.9		0.9456		0.9456		10.71 / 1.73	10.67 / 2.29		0.4006			0.5535	
*M-gH*	8.9 / 1.35	9.19 / 1.48		0.207		0.3549		9.07 / 1.41	9.35 / 1.69		0.344			0.516	
*Basal*	43.49 / 4.93	46.02 / 4.39		0.0016		0.0096*		45.19 / 4.85	45.72 / 3.53		0.4993			0.6307	
*Sinus*	46.93 / 6.93	49.75 / 5.6		0.0159		0.0477		49.01 / 6.21	48.16 / 5.38		0.6162			0.7214	
*STJ*	44.15 / 7.65	46.92 / 6.21		0.0136		0.0466		46.04 / 6.66	46.45 / 7.83		0.9281			0.9456	
*AA - Peri*	42.33 / 11.38	48.31 / 8.94		0.001		0.008*		46.56 / 10.2	46.2 / 9.42		0.9009			0.9401	
*AA - Length*	37.7 / 8.19	44.7 / 9.55		0.0002		0.0019*		42.42 9.58	43.81 / 10.58		0.7414			0.8088	
	**Visual hint AA aneurysm** ^**W**^							**Visual AV coaptation** ^**W**^							
***(M / SD)***	***Present***	***Absent***		***p***		***p adj.***		***Present***	***Absent***		***p***			***p adj.***	
*M-eH*	5.49 / 0.88	5.49 / 0.88		0.1526		0.293		5.7 / 1.01	5.72 / 0.77		0.6025			0.7214	
*M-cH*	11.11 / 1.69	11.11 / 1.69		0.1072		0.2262		10.73 / 1.8	10.54 / 1.9		0.3258			0.5045	
*M-gH*	9.26 / 1.47	9.26 / 1.47		0.4167		0.5556		9.18 / 1.46	8.5 / 1.22		0.0465			0.1116	
*Basal*	46.75 / 4.29	46.75 / 4.29		0.0102		0.0402		45.47 / 4.79	43.62 / 3.53		0.1405			0.281	
*Sinus*	52.86 / 4.77	52.86 / 4.77		*p* < 0.0001		0.0012*		49.04 / 6.06	47.86 / 6.92		0.4036			0.5535	
*STJ*	51.19 / 5.85	51.19 / 5.85		*p* < 0.0001		0.0012*		46.18 / 6.78	45.39 / 7.06		0.5316			0.6543	
*AA - Peri*	52.76 / 10.76	43.09 / 7.73		*p* < 0.0001		0.0012*		46.48 / 9.69	46.81 / 13.04		0.7285			0.8088	
*AA - Length*	50.13 / 8.27	39.04 / 8.17		*p* < 0.0001		0.0012*		42.97 / 9.01	39.72 / 13.95		0.0923			0.211	
	**Cusp fusion** ^**W**^							**Fenestration towards commissure** ^**W**^							
***(M / SD)***	***Present***	***Absent***		***p***		***p adj.***		***Present***	***Absent***		***p***			***p adj.***	
*M-eH*	5.51 / 0.8	5.72 / 1		0.6797		0.7768		5.16 / 0.45	5.75 / 1.01		0.0155			0.0477	
*M-cH*	11.3 / 1.57	10.65 / 1.82		0.195		0.3549		10.21 / 1.11	10.76 / 1.86		0.2506			0.4148	
*M-gH*	9.64 / 1.43	9.05 / 1.44		0.2063		0.3549		9.45 / 1.29	9.07 / 1.46		0.3869			0.5535	
*Basal*	45.84 / 3.46	45.20 / 4.8		0.4727		0.6132		45.5 / 3.68	45.23 / 4.79		0.7905			0.8432	
*Sinus*	52.2 / 4.77	48.56 / 6.19		0.0268		0.0757		51.88 / 4.74	48.60 / 6.21		0.0327			0.0826	
*STJ*	49.9 / 6.12	45.70 / 6.76		0.0327		0.0826		50.15 / 4.45	45.67 / 6.87		0.0109			0.0402	
*AA - Peri*	54.15 / 10.38	45.73 / 9.75		0.0029		0.0139		53.36 / 8.04	45.81 / 10.02		0.0046			0.0201	
*AA - Length*	50.6 / 10.83	41.78 / 9.22		0.0027		0.0139		51.03 / 8.33	41.74 / 9.42		0.0013			0.0089*	
	**Cause of death** ^**KW**^														
***(M / SD)***	***Unclear***		***Cardio-vascular***		***Intoxication***			***Non-cardiac disease***		***Traumatic***			***p***		
*M-eH*	5.78 / 1.06		5.57 / 0.92		6.25 / 0.79			5.58 / 1.01		5.74 / 1.03			0.1659 ^a^		
*M-cH*	10.13 / 1.15		10.91 / 2.53		10.95 / 1.19			10.53 / 1.64		10.75 / 1.41			0.3547 ^a^		
*M-gH*	9.08 / 1.92		9.14 / 1.65		9.43 / 1.33			9.02 / 1.11		9.06 / 1.43			0.9562 ^a^		
*Basal*	45.36 / 5.15		46.2 / 4.13		41.88 / 4.36			45.88 / 5.38		44.81 / 4.25			0.0854 ^a^		
*Sinus*	48.23 / 4.01		50.24 / 5.76		44.04 / 5.91			49.58 / 5.99		48.66 / 6.63			0.0752 ^a^		
*STJ*	45.12 / 3.72		48.21 / 7.05		41.21 / 5.21			45.96 / 6.05		45.88 / 7.52			0.0534 ^a^		
*AA - Peri*	44.55 / 6.3		49.65 / 9.4		35.79 / 11.33			47.82 / 8.68		46.12 / 10.36			0.003*^b^		
*AA - Length*	45.05 / 10.09		45.33 / 11.06		37.3 / 7.91			42.07 / 7.46		41.42 / 9.8			0.1687 ^a^		

The asterisks (*) highlight comparisons with *p* < 0.01. **Superscriptions**: ^a^ indicates that no post-hoc testing was performed; ^b^ indicates that post-hoc testing was performed and did not yield p-values < 0.01. **Abbreviations**: AA – tubular ascending aorta; AV – aortic valve; eH– effective height; gH – geometric height; HW– heart weight; KW – Kruskal-Wallis test; LV – left ventricle / left ventricular; M - mean; p adj.– p adjusted; SD – standard deviation; STJ – sino-tubular junction; W – Wilcoxon-test

### LV rigor mortis and AoR geometry

Comparative testing showed a smaller basal ring perimeter in cases with LV rigor mortis (M 43.49 mm – SD 4.93) compared to those without (M 46.02 mm – SD 4.39; p adjusted = 0.0096). In addition, AA perimeter and length were smaller in those with LV rigor mortis (AA length: M 37.7 mm – SD 8.19; AA perimeter: M 42.33 mm– SD 11.38) than in those without (AA length: M 44.7 mm– SD 9.55 – p adjusted = 0.0019; AA perimeter: M 48.31 mm – SD 8.94 – p adjusted = 0.008). The Sinus (p adjusted = 0.0477) and STJ (p adjusted = 0.0466) perimeter were comparable in cases with and without LV rigor mortis. Comparing the two groups, no differences regarding the mean eH (p adjusted = 0.2262), mean gH (p adjusted = 0.3549), and mean cH (p adjusted = 0.9456) were observed (Table 4).

### PMI and AoR

No differences between parameters describing aortic root geometry were observed when comparing cases with mild and moderate putrefaction (Table 4). Weak and mainly positive correlations between the PMI and all parameters describing the AoR geometry were observed (basal: ρ 0.089 / *p* = 0.2959; sinus: ρ 0.0515 / *p* = 0.5459; STJ: ρ 0.0792 / *p* = 0.3521; AA perimeter: ρ 0.1304 / *p* = 0.1245; AA length: ρ 0.1208 / *p* = 0.155; mean eH: ρ 0.0171 / *p* = 0.8412; mean gH: ρ 0.0325 / *p* = 0.7031; mean cH: ρ -0.1041 / *p* = 0.221, Fig. 4).

### Relationship of the AoR geometry measures among each other

The mean eH showed weak correlations with one of the AoR geometry parameters (basal: ρ -0.1287 / *p* = 0.1296; sinus: ρ -0.1342 / *p* = 0.114; STJ: ρ -0.1578 / *p* = 0.0626; AA perimeter: ρ -0.2017 / *p* = 0.0168; AA length: ρ -0.1549 / *p* = 0.0676; mean gH: ρ -0.0075 / *p* = 0.9296; mean cH: ρ 0.1473/ *p* = 0.0824). The mean cH showed positive correlations with the basal (ρ 0.2956 / *p* = 0.0004), sinus (ρ 0.2585 / *p* = 0.0020), and AA (ρ 0.2615 / *p* = 0.0018) perimeter, as well as with the mean gH (ρ 0.572/ *p* < 0.0001). Additionally, positive correlations of the mean cH were observed with the STJ perimeter (ρ 0.2122 / *p* = 0.0119), AA length (ρ 0.1828 / *p* = 0.0306), and mean eH (ρ 0.1473 / *p* = 0.0824). For the mean gH, positive correlations with the perimeters of the basal ring (ρ 0.3748 / *p* < 0.0001), the sinus (ρ 0.2926 / *p* = 0.0005), the STJ (ρ 0.2564 / *p* = 0.0022), and the AA (ρ 0.2282 / *p* = 0.0067) were observed. Between mean gH and AA length (ρ 0.1394/ *p* = 0.1005) or the mean eH (ρ -0.0075 / *p* = 0.9296) weaker correlations were found. The basal perimeter showed positive correlations with the perimeters of the sinus (ρ 0.7038 / *p* < 0.0001), the STJ (ρ 0.6649 / *p* < 0.0001), and the AA (ρ 0.5432 / *p* < 0.0001), as well as with the AA length (ρ 0.3479 / *p* = 0.0003), the mean gH (ρ 0.3748 / *p* < 0.0001), and the mean cH (ρ 0.2956/ p 0.0004). A weaker correlation between the basal perimeter and the mean eH (ρ -0.1287 / *p* = 0.1296) was observed. Positive correlations between the sinus and basal perimeter (ρ 0.7038 / *p* < 0.0001), the STJ perimeter (ρ 0.9412/ *p* < 0.0001), AA perimeter (ρ 0.7521/ *p* < 0.0001) and length (ρ 0.5586/ *p* < 0.0001), as well as with the mean gH (ρ 0.2926/ *p* = 0.0005) and the mean cH (ρ 0.2585/ *p* = 0.002) were observed. A weaker negative correlation between the sinus perimeter and the mean eH (ρ -0.1342/ *p* = 0.114) was found. Regarding the STJ perimeter, positive correlations with the basal perimeter (ρ 0.6649 / *p* < 0.0001), sinus perimeter (ρ 0.9412 / *p* < 0.0001), AA perimeter (ρ 0.7864 / *p* < 0.0001) and length (ρ 0.5725 / *p* < 0.0001), and mean gH (ρ 0.2564/ *p* = 0.0022) were observed. A weaker positive correlation between the STJ perimeter and the mean cH (ρ 0.2122/ *p* = 0.0119) was found, whereas a negative correlation between the STJ perimeter and the mean eH (ρ -0.1578/ *p* = 0.0626) was encountered. Regarding the AA perimeter, positive correlations with the basal perimeter (ρ 0.5432 / *p* < 0.0001), sinus perimeter (ρ 0.7521 / *p* < 0.0001), STJ perimeter (ρ 0.7864/ *p* < 0.0001), AA length (ρ 0.6803/ *p* < 0.0001), mean gH (ρ 0.2282/ *p* = 0.0067), and the mean cH (ρ 0.2615/ *p* = 0.0018) were found. A weaker negative correlation between the AA perimeter and the mean eH (ρ -0.2017 / *p* = 0.0168) was observed. Concerning the AA length, positive correlations with the basal perimeter (ρ 0.3479/ *p* < 0.0001), sinus perimeter (ρ 0.5586 / *p* < 0.0001), STJ perimeter (ρ 0.5725 / *p* < 0.0001), and AA perimeter (ρ 0.6803 / *p* < 0.0001) were found. Weaker positive correlations between the AA length and the mean gH (ρ 0.1394 / *p* = 0.1005) and the mean cH (ρ 0.1828/ *p* = 0.0306) were encountered. A negative correlation between AA length and the mean eH (ρ -0.1549 / *p* = 0.0676) was found.

### Factors potentially influencing postmortem AoR geometry

#### Correlation analysis

The correlation matrix encompassed the age, body weight, HW, MV perimeter, LV, and IVS thickness as potential influential factors. A summary of the analysis is given, details can be found in Fig. 4 and Supplemental File V: Weak correlations between any of these potential influencing factors and the mean gH, mean cH, or mean eH were observed. Regarding age, positive correlations with the basal perimeter (ρ 0.3001/ *p* = 0.0003), sinus perimeter (ρ 0.6278 / *p* < 0.0001), STJ perimeter (ρ 0.6414 / *p* < 0.0001), AA perimeter (ρ 0.7246/ *p* < 0.0001), and AA length (ρ 0.6549 / *p* < 0.0001) were found. I.e., older individuals presented with larger aortic perimeters and AA length. No signs of strong interference of the body weight with any of the AoR root geometry parameters were found. The weight of the heart correlated with the basal perimeter (ρ 0.2508 / *p* = 0.0028), sinus perimeter (ρ 0.3024 / *p* = 0.0003), STJ perimeter (ρ 0.3236 / *p* < 0.0001), AA perimeter (ρ 0.2569/ *p* = 0.0022), and length (ρ 0.2609/ *p* = 0.0019). Regarding the MV perimeter, positive correlations with the basal perimeter (ρ 0.4184 / *p* < 0.0001), sinus perimeter (ρ 0.5153 / *p* < 0.0001), STJ perimeter (ρ 0.5278 / *p* < 0.0001), AA perimeter (ρ 0.4679 / p 0.2868), and length (ρ 0.3459 / p 0.0762) were encountered. Both LV and IVS thickness showed weak correlations with the AoR geometry measures.

#### Comparative analysis

In the comparative analysis, the cause of death, and presence/absence of the following findings were assessed: cusp fusion, fenestrations extending towards a commissure, visual AV coaptation, and signs of an AA aneurysm. A summary of the results is presented (Table 4). No comparisons regarding AV coaptation showed p-values < 0.01. Regarding the cause of death, KW showed *p* < 0.01 for AA perimeter only; post-hoc testing showed no *p* < 0.01. In terms of the presence/absence of fused cusps, no differences regarding the AoR geometry parameter were found (p-values see Table 4). Regarding the presence/absence of fenestrations extending towards a commissure, cases with and without such fenestrations were comparable regarding mean eH, mean cH, mean gH, basal perimeter, sinus perimeter, STJ and AA perimeter. Longer AA length (M 51.03 mm – SD 8.33; p adjusted = 0.0089) was observed in cases with such fenestrations compared to those without (AA length: M 41.74 mm – SD 9.42). In cases with signs of an AA aneurysm, a larger sinus perimeter (M 52.86 mm – SD 4.77 vs. M 47.02 mm – SD 5.84; p adjusted = 0.0012), STJ perimeter (M 51.10 mm– SD 5.85 vs. M 43.47 mm – SD 5.82; p adjusted = 0.0012), AA perimeter (M 52.76 mm – SD 10.76 vs. M 43.09 mm – SD 7.73; p adjusted = 0.0012), and length (M 50.13 mm – SD 8.27 vs. M 39.04 mm – SD 8.17 mm; p adjusted = 0.0012) was found. Cases with and without signs of an AA aneurysm were comparable regarding the basal perimeter, mean gH, mean cH, and mean eH (p-values see Table 4).

### Summary of the main findings

The main findings of the present study are summarized as follows: (1) Hearts with LV rigor mortis present with a markedly smaller basal ring perimeter than those without. (2) Although comparably weak (*p* > 0.01), there were positive correlations between the PMI duration and the AoR perimeters, i.e. basal, sinus, and STJ perimeter. (3) Weak correlations (*p* > 0.01) between the mean eH, AoR, and AA dimensions were observed. (4) Positive correlations (*p* < 0.01) of the mean gH and mean cH with the AoR and AA measures were observed. (5) AA length showed positive correlations with AoR perimeters (Fig. 4; Table 4, and Supplemental File V).

## Discussion

SCD remains a concern not only for Legal Medicine but in general [36] as well as a medical and social challenge [36, 37] as it is responsible for approximately 4–5 million deaths per year worldwide [38]. As a result, there have been recent demands for multidisciplinary measures, including claims to improve autopsy rates worldwide to better identify SCD cases [36] that meet clinical and postmortem guidelines and recommendations (e.g [5, 8, 9, 39]). Although an autopsy can be considered the ‘gold standard’ for determining the cause of death [40], there are cases with macroscopically unremarkable hearts despite SCD [41]. In such cases, subsequent analyses like a molecular autopsy [41] or toxicological analyses [5] can help to identify the cause of death. However, such analyses are very time-consuming whereas the jurisdiction and law enforcement usually benefit from a fast initial (preliminary) expert opinion. Thus, certain morphological substrates of SCD (e.g [5])., are crucial for Legal Medicine experts. Considering discrepancies between clinical and postmortem studies, functional AV disease caused by changes in AoR geometry may be a currently imperceptible surrogate of SCD that could potentially be addressed by translational application of clinical anatomy knowledge and, once thoroughly established, could support swift and time-efficient SCD death diagnosis in a postmortem Legal Medicine setting in the future.

Cardiac surgeons, however, faced a similar problem and developed methods to intraoperatively assess AoR geometry, i.e. without circulation. Amongst others, the measurement of eH was established, for example, to enable AV assessment after aortotomy [29–32]. By assessing the eH-concept in a postmortem Legal Medicine setting the present study aligns with recent requests, for example, of the Swiss Society of Legal Medicine, to connect the clinical and postmortem sectors to further improve diagnostic performance [3]. The long-term goal of subsequent studies will be to establish AoR geometry as a diagnostic tool in the postmortem setting, in particular Legal Medicine. Further, potentially applicable methods for functional assessment of AV function in a postmortem environment could include perfusion methods [42, 43].

### Peculiarities of the autopsy setting

There are, however, relevant differences between the clinical and postmortem settings. For example, postmortem imaging studies demonstrated that the aortic shape and diameters change after death [44], likely due to the missing circulation. Additionally, the postmortem sector is confronted with LV rigor mortis and putrefaction, which presumably affect the AoR geometry. In contrast, one could assume that the cardioplegic arrest during cardiac surgery provides somehow comparable circumstances. In the present study particular attention was paid to the rigor mortis of the LV, as basic science provides evidence that rigor mortis may be different in skeletal muscle and myocardium.

Experiments (e.g [45, 46]). suggest that rigor mortis of the skeletal muscle is related to missing adenosine triphosphate (ATP) (for example [47]), as binding of ATP to myosin releases the myosin from the actin [48]. Although both the myocardium and the skeletal muscle are striated muscles, they differ in certain ways [48]. For example, in skeletal muscle and myocardium, ATP hydrolysis is the basis for a repeated cross-bridge cycle [48]. In the myocardium, it was found that adenosine diphosphate (ADP) can also trigger a contraction [49]. To our knowledge, comparable findings are not known for the skeletal muscle. Based on these observations, the authors assume that the myocardium not only exhibits ATP-induced cadaveric rigidity but also ADP-induced contraction to some degree. With the myocardium extending into the AoR [50], especially in congenital AV malformation [51], this would presumably lead to changes in the AoR geometry, especially the basal ring and the aorto-ventricular junction. These assumptions are supported by the findings of the present study. Hearts with LV rigor mortis presented with smaller basal rings (p adjusted < 0.01) compared to hearts without. In contrast, the other AoR measurements were comparable between hearts with and without cadaveric rigidity (Table 4). This at first glance contradictory finding could be explained by the fact that the influence of LV rigidity and AoR geometry is limited to the basal ring, as the aorto-ventricular junction does not extend further upward. Not only do such phenomena interfere with cardiac geometry, but also with postmortem organ processing. For example, the fixation of the heart affects the dimensions of the heart [52]. For this reason, only non-fixated hearts were examined in the present study. Given the differences between the clinical and postmortem setting, the present study does not yet al.low for the assessment of eH and the other AoR measures as a diagnostic tool. For example, due to the differences described above, the clinical cut-off values (e.g. an eH of about 9 mm can be considered normal if the gH is about 20 mm [32]) must be re-evaluated and validated in the postmortem setting. The results of the present study emphasize that caution is required and that further investigations must be carried out. Thus, despite comparatively weak correlations (*p* > 0.01), positive correlations were found between the PMI and the AoR measures, while at the same time, negative correlations were found between the mean eH and the AoR perimeters.

These results can be considered as proof of concept, as even postmortem dilatation leads to a decrease in eH, which is consistent with what is known in the clinical setting [19, 20]. Here, dilatation of the AoR and the AA [17, 19, 20] was associated with impaired AV function and a reduction in eH.

### Dimensions of the tubular ascending aorta

In the past, thresholds for AA dimensions were set in the clinical setting, e.g. AA replacement was recommended if the AA exceeded a diameter of 4 cm in BAV patients [53]. Nowadays, the impact of body size (i.e. height, weight, etc.) is given more consideration. For example, normalization methods (e.g [33]). or approaches to calculate an upper limit of what is normal for a given patient (e.g [54]). are used. However, not only the AA diameter is dependent on body size, but also the gH [31]. At the same time, gH and eH are interrelated [32]. For this reason, all AoR geometry measures were normalized to body height in the present study. With this approach, inter-individual variances due to differences in body size as potential mediator were minimized.

In addition to AA diameter, AA length is also clinically important. Usually, the AA elongates with aging [55], but also in the context of BAV [56]. AA elongation has been associated with an increased risk of AA dissection, a complication with high mortality [57]. Despite its clinical importance, the authors have not been able to find a postmortem study on AA length. Therefore, AA length was included in the present study. As a result, the positive correlation between AA length and age could be reproduced in the present study. Furthermore, despite normalization for height, length showed a positive correlation with the AoR perimeters. Thus, AA length seems to be somewhat related to aortic size in general, and AoR geometry in particular.

### Limitations

Besides the limitations discussed this far, the following limitations of the present study must be pointed out: (1) Due to the exploratory nature of the present study, multiple p-values were computed. This can be associated with a cumulating α-fault [58]. To address this, a significance level of α = 0.01 was chosen and p adjustment for the comparative analysis was applied. Such an approach may, however, be associated with overlooking smaller, but relevant differences, resulting in p-values > 0.01. (2) Despite advances in rigorous data management that allow for normalization, such an approach limits insights into variance and artificially lowers the case number, which in turn reduces statistical power. (3) Although it was shown that PMI and LV rigor mortis affect AoR geometry, it was not possible to determine how long after death AoR geometry measures can be considered ‘stable’ due to the study design (i.e. exclusion of cases with severe putrefaction). (4) For the sake of clarity, this first exploratory study focused on mean gH, mean eH, and mean cH. By that, we were able to compare all individuals, as no missing values remained after data preparation. Information on how the different cusps and commissures might differ is lost with this approach. Therefore, further studies are needed to investigate the individual cusps and commissures in detail. (5) In this study, special emphasis was placed on AoR geometry. It was neglected that all the heart valves are connected to some degree by the cardiac skeleton [59]. Due to direct aortomitral continuity [60], likely, both post- and antemortem, artificial (postmortem), and pathologic (antemortem) changes in the MV could affect postmortem AoR geometry. Therefore, further studies are needed to evaluate postmortem MV and AoR geometry in conjunction with each other.

## Conclusion

The present study is the first to assess postmortem AoR geometry including eH (see Supplemental File I – Appendix B). The results suggest that although postmortem features affect AoR geometry, it may be possible to apply the knowledge on clinical anatomy of the AoR to postmortem diagnostic panel. As clinicians continually work to improve their understanding of AoR geometry (e.g [61])., the potential of such a translational approach seems to be growing. With the findings that postmortem phenomena, especially LV rigor mortis, influence the AoR geometry, systematic studies further investigating this translational approach may identify how such morphometric parameters, like the eH, can be integrated in postmortem expert opinions in the future. This would support swift and time-efficient diagnoses in Legal Medicine frequently involving sudden unexpected deaths and thus, cases of SCD.

## Electronic supplementary material

Below is the link to the electronic supplementary material.

Supplementary Material I: Appendix A - Abbreviations; Appendix B - Systematic literature search; Appendix C - Cause of Death; Appendix D - Further descriptive statistics.

Supplementary Material II: Anonymized data set.

Supplementary Material III: R code.

Supplementary Material IV: Comments on the data preparation.

Supplementary Material V: P-values and correlation coefficients complementing the correlation matrix (Fig. 4).

## Data Availability

An anonymized version of the data set underlying the present study can be found in Supplemental File II. Anonymization was needed due to data protection requirements. It was done according to German recommendations (https://stiftungdatenschutz.org/fileadmin/Redaktion/Dokumente/Anonymisierung_personenbezogener_Daten/SDS_Studie_Praxisleitfaden-Anonymisieren-Web_01.pdf) by interchanging rows and columns.

## Declarations

### Artificial intelligence statement

No generative artificial intelligence (AI) has been used for the preparation of the present manuscript. The AI-based language software *Grammarly* (v1.2.116.1536) was used in the preparation of the manuscript to assist with correct phrasing and wording.
