# Supplementary material for: Left ventricular rigor mortis interferes with postmortem aortic root geometry
Source: Int J Legal Med. 2025 Jan 21;139(3):1113–30. doi: 10.1007/s00414-025-03409-1 (PMC12003611; doi:10.1007/s00414-025-03409-1)

Supplemental File I - Appendices

Left ventricular rigor mortis interferes with postmortem aortic root geometry

Authors and Affiliations

Jan Michael Federspiel [1,*], Karen B. Abeln [2], Frank Ramsthaler [1], Thomas Tschernig [3], Peter H. Schmidt [1]

1. Institute for Legal Medicine, Saarland University, Faculty of Medicine, Campus Homburg, Homburg/Saar, Germany.

2. Department of Cardiac Surgery, Saarland University Medical Center, Homburg/Saar, Germany.

3. Institute of Anatomy, Saarland University, Faculty of Medicine, Campus Homburg, Homburg/Saar, Germany.

* **Corresponding Author:** Dr. med. Jan Michael Federspiel. Institute for Legal Medicine, Saarland University, Faculty of Medicine, Campus Homburg, Building 49.1, Kirrberger Straße 100, 66421 Homburg/Saar, Germany. E-Mail: jmfederspiel@outlook.com. Phone (secretary): 0049 6841 16 26304.

Appendix A – Abbreviations

AA	Tubular ascending aorta
ADP	Adenosinediphosphate
AV	Aortic valve
AoR	Aortic root
ATP	Adenosinetriphosphate
BAV	Bicuspid aortic valve
BMI	Body mass index
BSA	Body surface area
cH	Commissural height
eH	Effective height
gH	Geometric height
HW	Heart weight
IVS	Interventricular septum
KW	Kruskal Wallis test
LCC	Left coronary cusp
LV	Left ventricle / left ventricular
M	Mean
MV	Mitral valve
NCC	Left coronary cusp
PMI	Postmortem interval
RCC	Right coronary cusp
RV	Right ventricle / right ventricular
SCD	Sudden cardiac death
SD	Standard deviation
STJ	Sino-tubular junction
W	Wilcoxon test
ρ	Rho, i.e., Spearman correlation coefficient

Appendix B – Systematic literature search

Data base: PubMed.

Date: 29th June 2024 at 02:28 pm.

Advanced search algorithm: (((effective height) AND (aortic)) AND ((autopsy) OR (postmortem))).

The algorithm identified the following publications:

1. Sukhodolya T, Damjanovic D, Beyersdorf F, Benk C, Heilmann C, Blanke P, Euringer W, Trummer G. Standard intra-aortic counterpulsation balloon may cause temporary occlusion of mesenterial and renal arteries. ASAIO J. 2013 Nov-Dec;59(6):593-9. doi: 10.1097/MAT.0b013e3182a4b343. PMID: 24172264.
2. Schyma C, Hagemeier L, Madea B. Suicide by head explosion: unusual blast wave injuries to the cardiovascular system. Int J Legal Med. 2011 Jul;125(4):473-8. doi: 10.1007/s00414-010-0452-6. Epub 2010 May 12. PMID: 20461526.
3. Virmani R, Avolio AP, Mergner WJ, Robinowitz M, Herderick EE, Cornhill JF, Guo SY, Liu TH, Ou DY, O'Rourke M. Effect of aging on aortic morphology in populations with high and low prevalence of hypertension and atherosclerosis. Comparison between occidental and Chinese communities. Am J Pathol. 1991 Nov;139(5):1119-29. PMID: 1951629; PMCID: PMC1886332.
4. Watanabe S, Terazawa K, Matoba K. Age estimation from quantitative evaluation of atherosclerosis of abdominal aorta in Japanese. Hokkaido Igaku Zasshi. 2007 Mar;82(2):91-8. PMID: 17450946.
5. Ma L, Tozzi P, Huber CH, Taub S, Gerelle G, von Segesser LK. Double-crowned valved stents for off-pump mitral valve replacement. Eur J Cardiothorac Surg. 2005 Aug;28(2):194-8; discussion 198-9. doi: 10.1016/j.ejcts.2004.12.068. PMID: 15950482.
6. Pielmeier R, Engelke E, Legler M, Haist V, Hopster-Iversen C, Distl O. Kongenitale Herzanomalien (Fallotsche Pentalogie) bei einem zweijährigen Schafbock mit Brachygnathia inferior [Congenital cardiac anomalies (pentalogy of Fallot) in a two year old ram with brachygnathia inferior]. Berl Munch Tierarztl Wochenschr. 2013 May-Jun;126(5-6):256-63. German. PMID: 23758042.

None of these articles systematically assessed the effective height in a postmortem setting.

Appendix C – Cause of death

The following table provides a description of the exact cause of death in each of the 140 cases forwarded to the statistical analyses. 'Classification cause of death for statistical analyses' shows how the cause of death has been encoded on a nominal scale to allow for statistical analyses.

#	*Cause of death*	*Classification cause of death for statistical analyses*
1	Fall, fracture of the cervical spine	traumatic death
2	Rupture of the interventricular septum following myocardial infarction	cardio-vascular cause of death
3	Killing, several stab and cut injuries	traumatic death
4	Suspicion of myocarditis following vaccination	cardio-vascular cause of death
5	Coronary heart disease	cardio-vascular cause of death
6	Staircase fall, craniocerebral trauma	traumatic death
7	Septic multi-organ failure	non-cardiac disease
8	Myocardial infarction	cardio-vascular cause of death
9	Myocardial infarction	cardio-vascular cause of death
10	Intoxication	intoxication
11	Recurrent pulmonary artery thrombembolism	cardio-vascular cause of death
12	Freezing to death	traumatic death
13	Polytrauma	traumatic death
14	Pulmonary artery thrombembolism	cardio-vascular cause of death
15	Asphyxiation due to laryngeal carcinoma	non-cardiac disease
16	Macroscopically not reliably detectable	unclear
17	Killing, several stabs and cut injuries	traumatic death
18	Ischemic rupture of the interventricular septum following postoperative bypass-dissection	cardio-vascular cause of death
19	Craniocerebral trauma	traumatic death
20	Convulsive seizure	traumatic death
21	Killing, several stabs and cut injuries	traumatic death
22	Killing, headshot	traumatic death
23	Pericardial tamponade due to aortic rupture due to aortic dissection (Stanford Type A / DeBakey Type II)	cardio-vascular cause of death
24	Septic multi-organ failure	non-cardiac disease
25	Acute heart failure due to ischemia	cardio-vascular cause of death
26	Suicide, intentional drug overdosing	intoxication
27	traumatic asphyxiation	traumatic death
28	Septic multi-organ failure	non-cardiac disease
29	Septic multi-organ failure	non-cardiac disease
30	Craniocerebral trauma	traumatic death
31	Pulmonary artery thrombembolism	cardio-vascular cause of death
32	Septic multi-organ failure	non-cardiac disease
33	Pericardial tamponade due to ischemic rupture of the left ventricle	cardio-vascular cause of death
34	Pericardial tamponade due to aortic rupture due to aortic dissection (Stanford Type A / DeBakey Type I)	cardio-vascular cause of death
35	Acute on chronic heart failure due to acute on chronic myocardial ischemia	cardio-vascular cause of death
36	polytrauma	traumatic death
37	Pulmonary artery thrombembolism	cardio-vascular cause of death
38	Asphyxiation due to lung cancer	non-cardiac disease

39	Pericardial tamponade due to ventricular rupture	cardio-vascular cause of death
40	Suicide, Hanging	traumatic death
41	Septic multi-organ failure after a fall from great height	non-cardiac disease
42	Acute on chronic myocardial ischemia	cardio-vascular cause of death
43	Acute liver failure	non-cardiac disease
44	Intoxication (Heroin)	intoxication
45	Septic multi-organ failure	non-cardiac disease
46	Septic multi-organ failure	non-cardiac disease
47	Acute severe blood loss following detachment of a bypass graft	cardio-vascular cause of death
48	Intracerebral hemorrhage	cardio-vascular cause of death
49	traffic accident	traumatic death
50	Suspicion of excited delirium	non-cardiac disease
51	Oesophageal varices bleeding	non-cardiac disease
52	Intoxication	intoxication
53	Hemorrhagic shock due to intraoperative complications	traumatic death
54	Pneumonia	non-cardiac disease
55	Pulmonary artery thrombembolism	cardio-vascular cause of death
56	Macroscopically not reliably detectable	unclear
57	Endocarditis with aortic root abscess	cardio-vascular cause of death
58	Macroscopically not reliably detectable	unclear
59	Intoxication	intoxication
60	Suicide, sharp force against the neck	traumatic death
61	Drowning	traumatic death
62	Hit by a falling tree, craniocerebral injury	traumatic death
63	Pericardial tamponade due to ischemic rupture of the left ventricle	cardio-vascular cause of death
64	Pericardial tamponade due to aortic rupture due to aortic dissection (Stanford Type A / DeBakey Type I)	cardio-vascular cause of death
65	Craniocerebral trauma	traumatic death
66	Pneumonia and meningitis	non-cardiac disease
67	Macroscopically not reliably detectable	unclear
68	Macroscopically not reliably detectable	unclear
69	Bleeding from a duodenal ulcer	non-cardiac disease
70	polytrauma	traumatic death
71	Craniocerebral trauma	traumatic death
72	Ventricular fibrillation during pneumectomy	cardio-vascular cause of death
73	Macroscopically not reliably detectable	unclear
74	Macroscopically not reliably detectable	unclear
75	Suicide, Shot into the stomach	non-cardiac disease
76	gastro-intestinal hemorrhage	non-cardiac disease
77	polytrauma	traumatic death
78	Suicide, Shot through thorax and abdomen	traumatic death
79	Acute heart failure during induction of anesthesia of a patient with severe chronic heart failure	cardio-vascular cause of death
80	gastro-intestinal hemorrhage	non-cardiac disease
81	Pulmonary artery thrombembolism	cardio-vascular cause of death
82	Septic multi-organ failure	non-cardiac disease
83	Septic multi-organ failure	non-cardiac disease
84	Pneumonia following car accident	non-cardiac disease

85	Intoxication	intoxication
86	Polytrauma	traumatic death
87	Acute on chronic heart failure	cardio-vascular cause of death
88	Macroscopically not reliably detectable	unclear
89	Pneumonia	non-cardiac disease
90	Suicide, headshot	traumatic death
91	Pulmonary artery thrombembolism	cardio-vascular cause of death
92	Intoxication	intoxication
93	Suicide, polytrauma	traumatic death
94	Intracerebral hemorrhage	cardio-vascular cause of death
95	Craniocerebral trauma	traumatic death
96	Asphyxiation	non-cardiac disease
97	Intracranial hemorrahge due to rupture of an aneurysm of the anterior cerebral artery	cardio-vascular cause of death
98	Acute on chronic heart failure	cardio-vascular cause of death
99	Septic multi-organ failure	non-cardiac disease
100	Pneumonia	non-cardiac disease
101	Hemorrhagic shock due to intraoperative complications	traumatic death
102	Suicide, hanging	traumatic death
103	Severe liver cirrhosis, acute hydropic decompensation	non-cardiac disease
104	polytrauma	traumatic death
105	Pneumonia	non-cardiac disease
106	Suicide, Mirtazapin overdose	intoxication
107	Macroscopically not reliably detectable	unclear
108	Hypoxic brain damage, likely due to sudden circulatory arrest due to severely damaged heart	cardio-vascular cause of death
109	polytrauma	traumatic death
110	polytrauma	traumatic death
111	Craniocerebral trauma	traumatic death
112	Hemorrhagic shock following an accident (fall)	traumatic death
113	Suicide, headshot	traumatic death
114	Killing, headshot	traumatic death
115	Pneumonia and pleural empyema	non-cardiac disease
116	Hemorrhagic shock due to rupture of a dialysis shunt due to shunt infection	non-cardiac disease
117	Septic multi-organ failure	non-cardiac disease
118	Recurrent myocardial infarction	cardio-vascular cause of death
119	Intoxication	intoxication
120	Freezing to death	traumatic death
121	Acute on chronic heart failure during surgery needed due to an accident (fall)	cardio-vascular cause of death
122	Pneumonia	non-cardiac disease
123	Intracerebral hemorrhage	cardio-vascular cause of death
124	Upper gastrointestinal bleeding and blood aspiration	non-cardiac disease
125	Intoxication	intoxication
126	Hanging	traumatic death
127	Macroscopically not reliably detectable	unclear
128	Suicide, fall from the height	traumatic death
129	Traumatic subdural hemorrhage	traumatic death

130	Macroscopically not reliably detectable	unclear
131	Intoxication	intoxication
132	An intramural course of the left anterior descending with acute myocardial infarction	cardio-vascular cause of death
133	Septic multi-organ failure	non-cardiac disease
134	Pulmonary artery thrombembolism	cardio-vascular cause of death
135	Myocardial infarction	cardio-vascular cause of death
136	Acute on chronic heart failure with a severely damaged heart	cardio-vascular cause of death
137	Killing, stab/cut injury of the ascending aorta	traumatic death
138	Intoxication	intoxication
139	Septic multi-organ failure	non-cardiac disease
140	Suicide, Fall from great height	traumatic death

Appendix D – Further descriptive statistics

In the following several violin plots (dark blue) with integrated box plots (white) are displayed complementing the descriptive statistics provided in the main manuscript. The violin plot (dark blue area) resembles the distribution of the respective parameter. The wider the area is, the more individuals showed the respective value. The box plot (white) displays the desprictive statistics. Each box covers the interquartile range of the respective parameter (i.e., the top shows 25th percentile, bottom shows 75th percentile). The line within the box resembles the median. Each whisker ends above or below the first and third quartile plus/minus 1.5 times the interquartile range. Values outside these borders are displayed as larger dots, i.e. outliers.

D.1 - Violin and boxplot- Measured and predicted heart weights

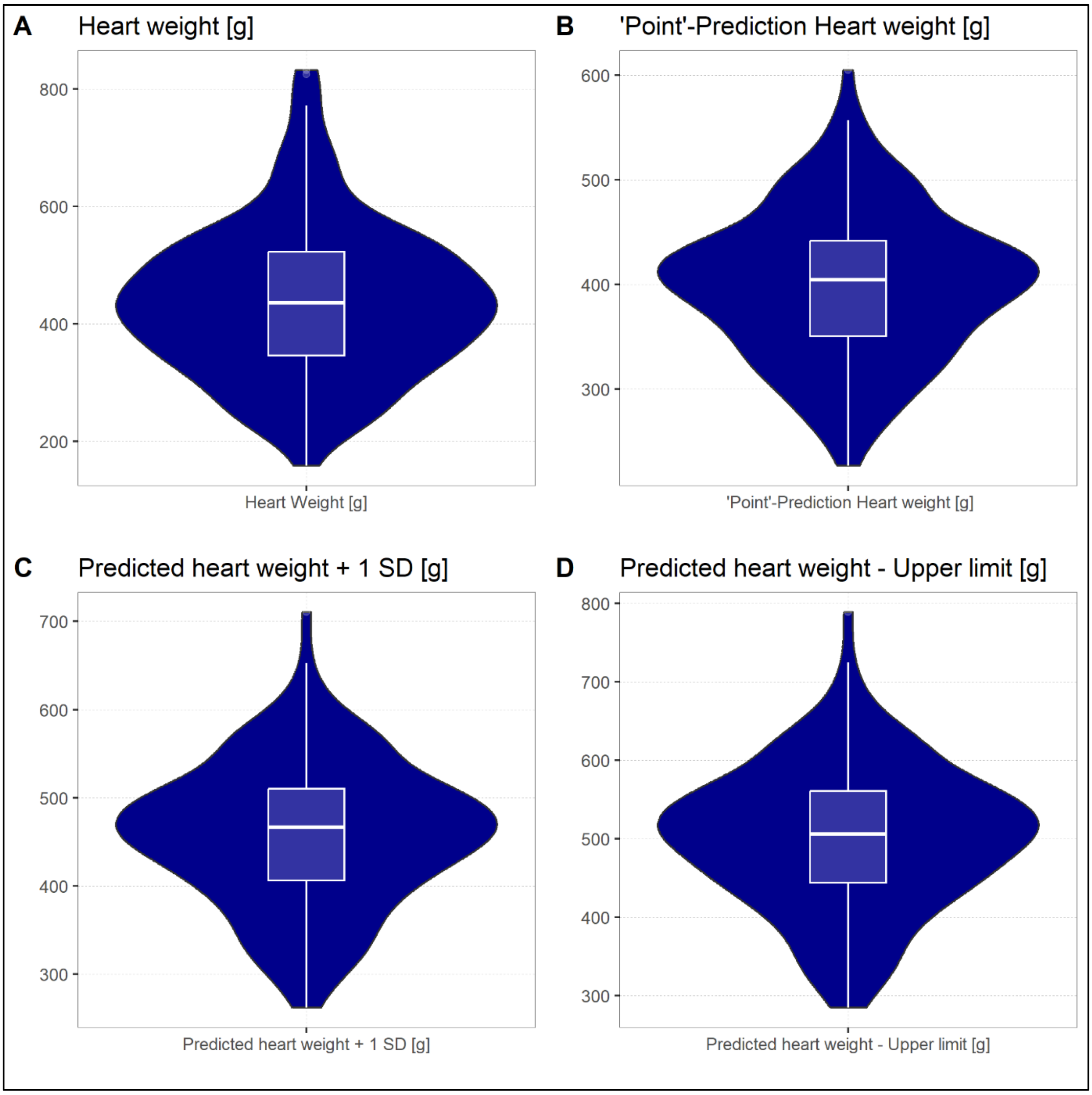

D.2 - Violin and boxplot - effective height measurements

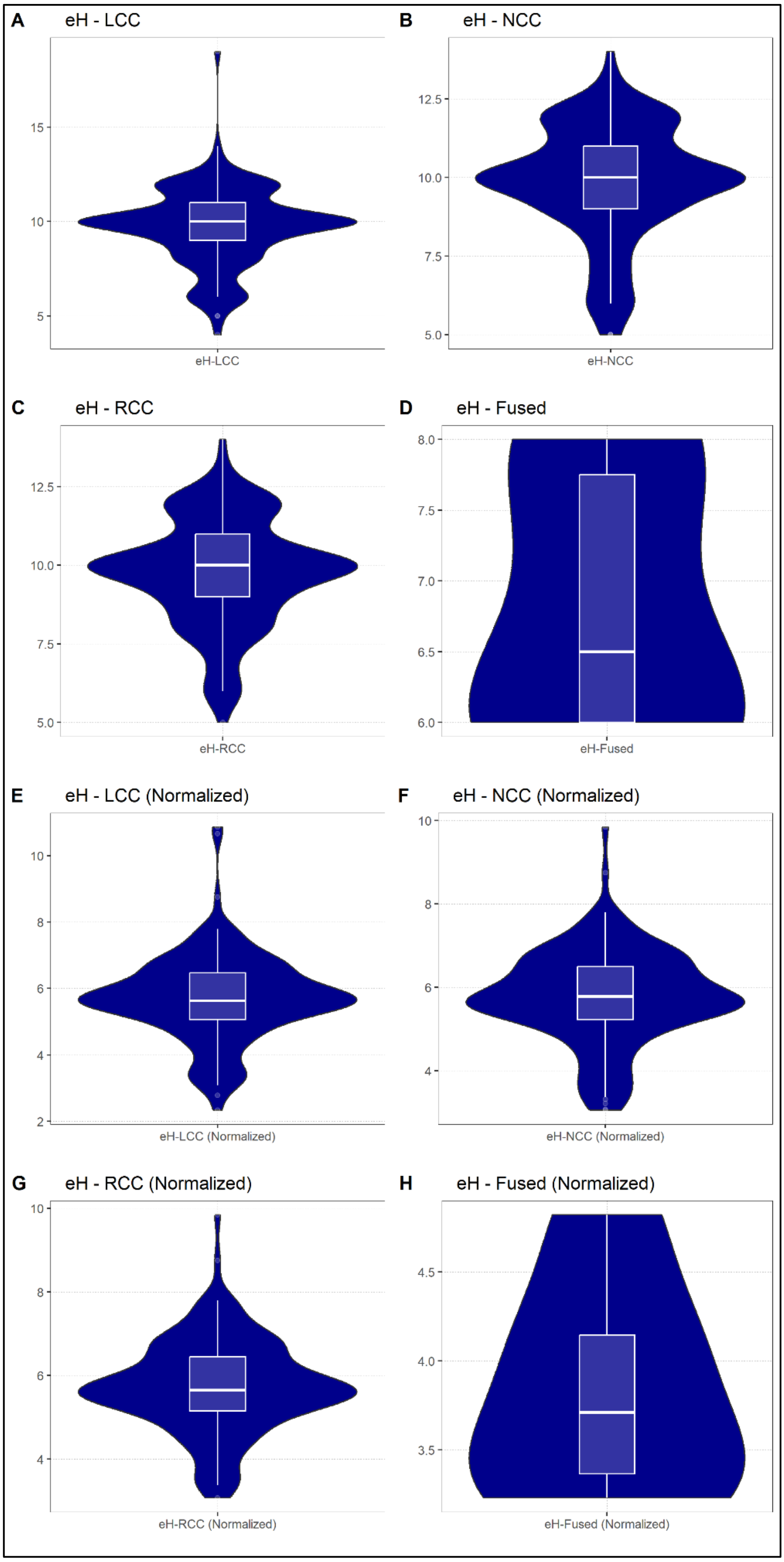

D.3 - Violin and boxplot - geometric height measurements

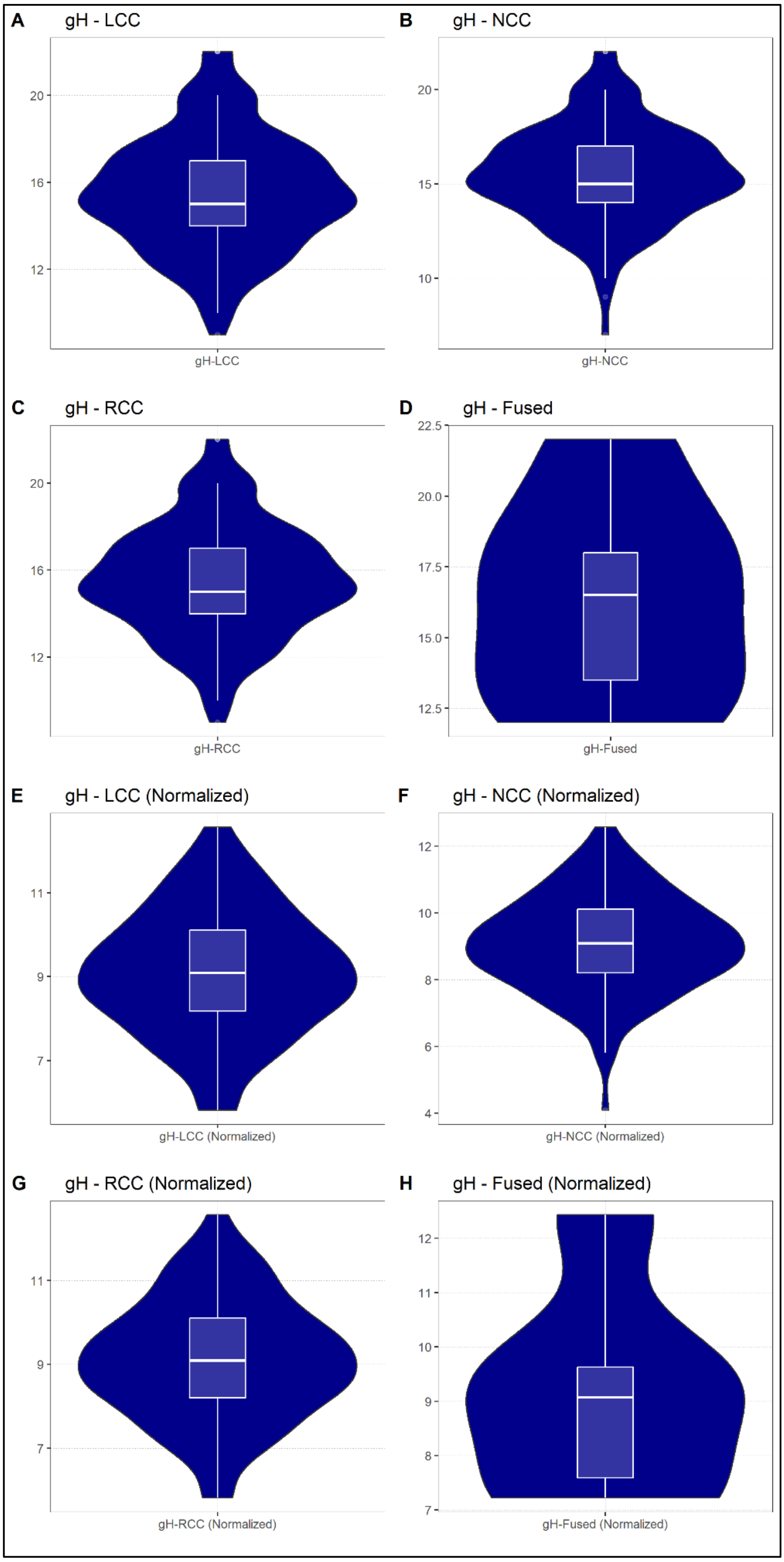

D.4 - Violin and boxplot - commissural height measurement

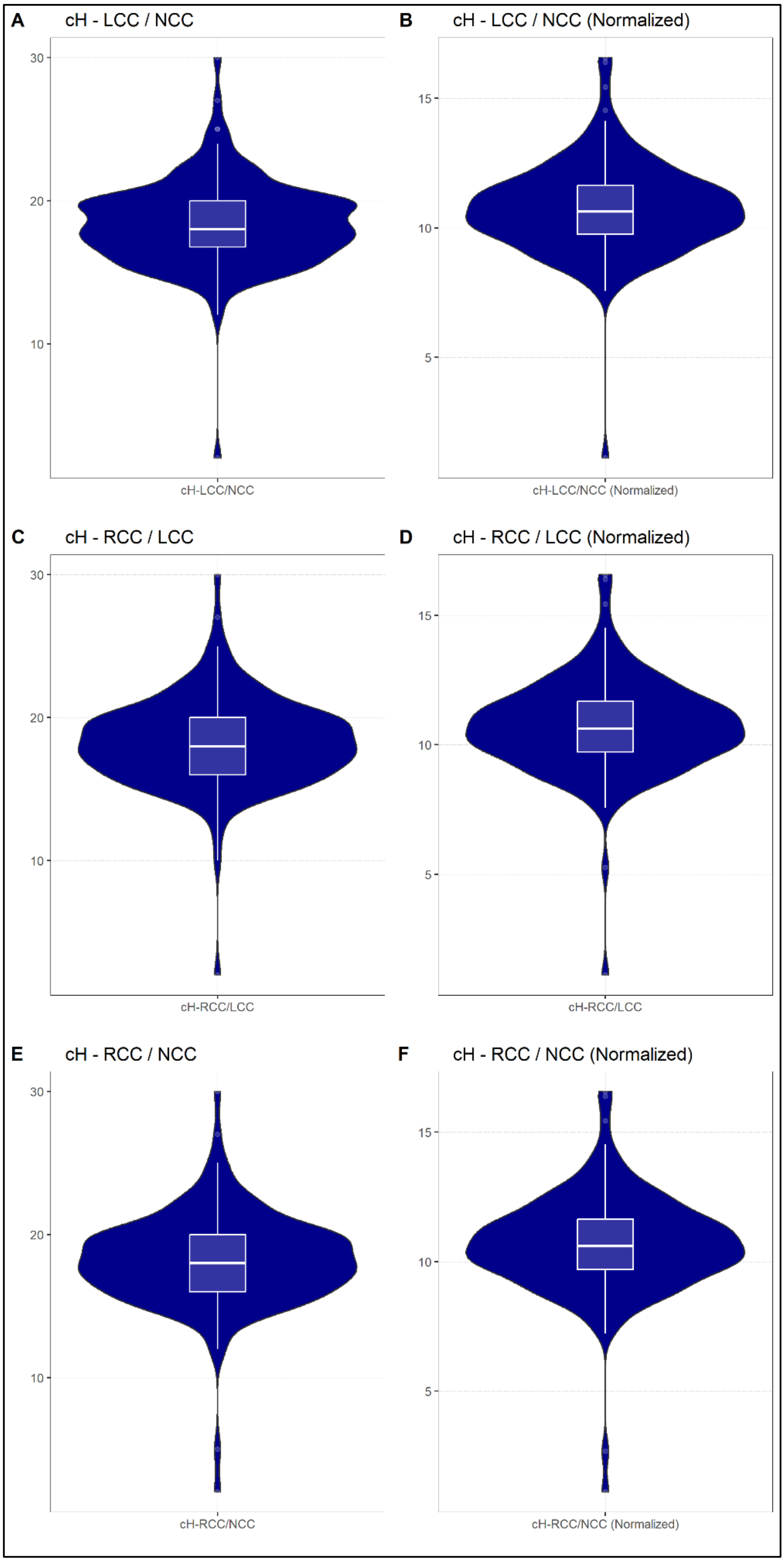

D.5 - Violin and boxplot - non-normalized aortic root measurements

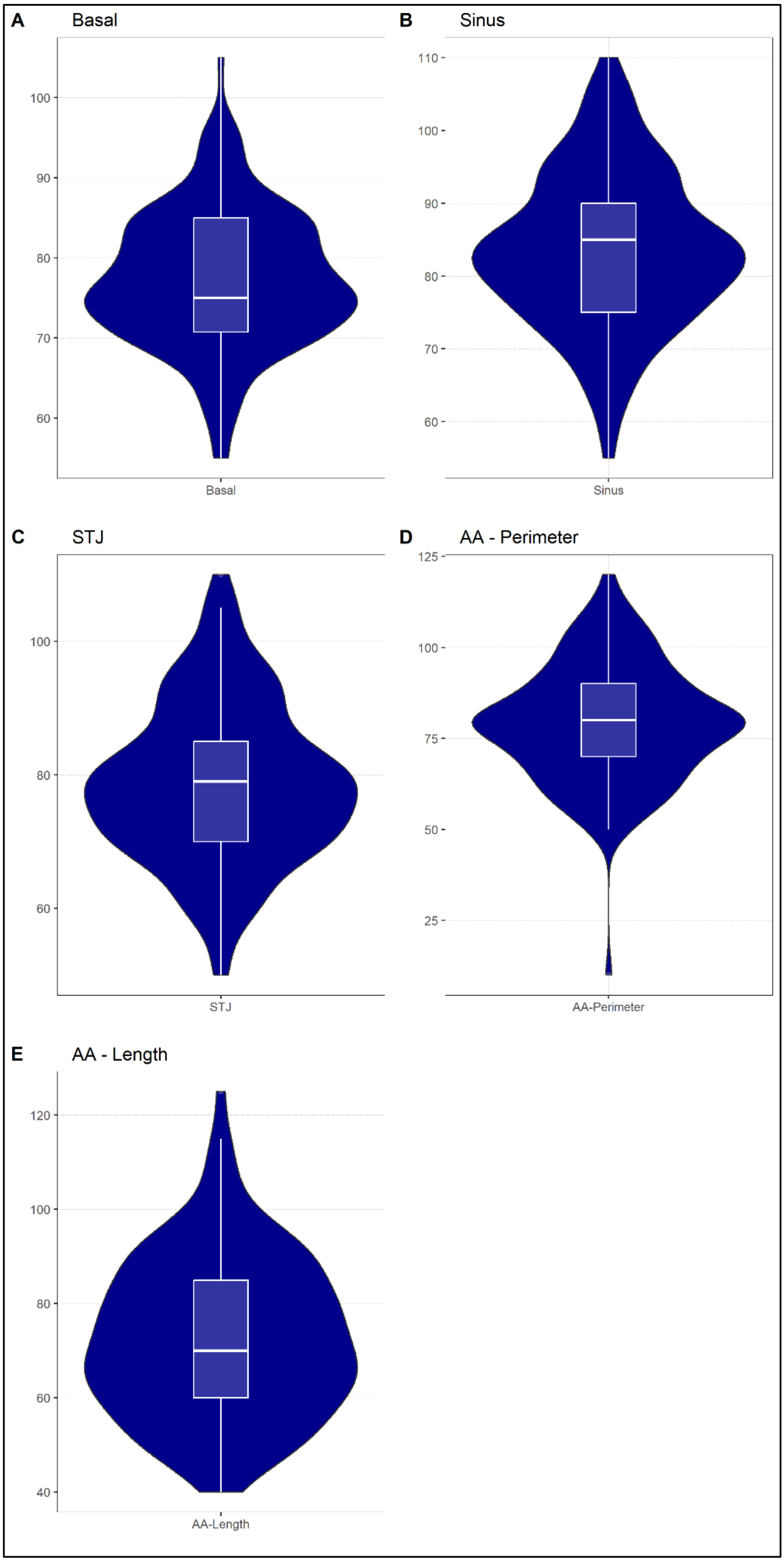

Supplement: Supplementary file 1 — Supplementary Material I: Appendix A - Abbreviations; Appendix B - Systematic literature search; Appendix C - Cause of Death; Appendix D - Further descriptive statistics. [file 414_2025_3409_MOESM1_ESM.pdf]
